# Design, Fabrication, and Testing of a 3D-Printed Model Rocket with Integrated Telemetry Systems

**DOI:** 10.3390/s26165022

**Published:** 2026-08-07

**Authors:** Philippos G. Moschidis, Petros S. Bithas, Florian Meyer

**Affiliations:** 1 Faculty of Production Engineering, University of Bremen, 28359 Bremen, Germany; 2 Department of Digital Industry Technologies, National and Kapodistrian University of Athens, 15772 Athens, Greece; pbithas@uoa.gr; 3 Center of Applied Space Technology and Microgravity (ZARM), University of Bremen, 28359 Bremen, Germany; florian.meyer@zarm.uni-bremen.de

**Keywords:** additive manufacturing, model rocketry, flight computer, telemetry, Raspberry Pi, embedded systems, OpenRocket, PETG, aerospace experimentation, flight-data acquisition

## Abstract

This study presents the design, fabrication, and experimental validation of the Hermes reusable model rocket platform integrating additive manufacturing, onboard sensing, and telemetry capabilities for low-cost aerospace experimentation. The rocket was manufactured using modular Polyethylene Terephthalate Glycol (PETG) components produced through fused filament fabrication to achieve a lightweight and structurally robust configuration suitable for repeated flight operations. A custom flight computer based on a Raspberry Pi Zero 2W was developed to acquire in-flight data from an inertial measurement unit, barometric pressure sensor, and Global Positioning System module, while an onboard camera enabled post-flight trajectory assessment. Aerodynamic performance and stability were evaluated using OpenRocket simulations, and propulsion was provided by a cluster of Klima D9-5 solid rocket motors. Four experimental flights were conducted to evaluate the integrated system architecture, assess telemetry and sensor performance, and compare experimental flight data with simulation predictions. The recorded measurements successfully captured the primary flight phases, including launch, ascent, apogee, descent, and recovery. The experimental results showed qualitative agreement with the simulated flight profiles; however, deviations in apogee altitude, acceleration, and flight duration were observed due to aerodynamic drag, environmental disturbances, motor-performance variability, and implementation-related limitations. The flight campaigns additionally identified practical challenges associated with wireless telemetry reliability, GPS signal acquisition, electronic protection, and parachute deployment, leading to iterative system improvements. From a sensing perspective, the flight campaigns demonstrate the operation and limitations of a low-cost embedded acquisition architecture under dynamic conditions, including the effects of sampling rate, sensor calibration, synchronization, wireless-link interruption, and local data preservation on the quality of the recorded flight measurements. The presented platform demonstrates the feasibility of combining low-cost additive manufacturing techniques with commercially available embedded electronics for reusable aerospace testing and educational applications. The proposed system further provides a flexible experimental framework for flight-data acquisition, simulation validation, and iterative development in academic and amateur rocketry research.

## 1. Introduction

### 1.1. Background and Motivation

Recent advances in additive manufacturing, embedded electronics, and low-cost sensing technologies have significantly expanded the capabilities of experimental and educational model rocketry. The integration of compact micro-controllers, inertial sensors, barometric altimeters, Global Positioning System (GPS) modules, and wireless telemetry systems has enabled the development of reusable aerospace platforms capable of acquiring real-time flight data with relatively low cost and complexity. In parallel, fused filament fabrication (FFF) has facilitated the rapid fabrication of lightweight and customisable rocket structures with reduced manufacturing constraints. These technological developments have increasingly transformed model rocketry from a primarily recreational activity into an accessible experimental platform for aerospace engineering research, sensor validation, flight-data acquisition, and systems integration. Universities, student teams, and amateur aerospace groups have adopted such platforms as low-cost testbeds for validating aerodynamic models, embedded avionics, telemetry architectures, and recovery systems prior to larger-scale implementation. Within this context, the Hermes Rocket Project was developed as a reusable experimental platform combining additive manufacturing, onboard sensing, and telemetry capabilities for flight-data acquisition and system validation [1].

### 1.2. Related Work

Recent research in model rocketry has increasingly focused on the integration of simulation tools, embedded avionics, and experimental flight validation. Rohini et al. [2] presented the design and experimental evaluation of a solid-propellant model rocket analyzed using OpenRocket software. Their work combined numerical simulation with experimental thrust characterization of a custom dextrose–potassium nitrate propellant, demonstrating the importance of integrating analytical and experimental methodologies during rocket development. Brown et al. [3] evaluated the accuracy of flight-prediction methods by comparing OpenRocket simulations with a custom six-degree-of-freedom (6DOF) simulation framework incorporating aerodynamic coefficients derived from computational fluid dynamics and wind-tunnel testing. To experimentally validate the simulated trajectories, the authors additionally developed the FASTsensor avionics system for onboard telemetry acquisition. Their results highlighted the importance of flight-data collection and experimental validation in improving trajectory-prediction accuracy. Low-cost flight-computer architectures have also received increasing attention in educational and amateur rocketry applications. Telaak et al. [4] proposed an Arduino-based avionics system integrating an inertial measurement unit (IMU), barometric sensor, and GPS module for telemetry and recovery applications. Similarly, Binz et al. [5] presented the open-source Vega flight computer developed by CATS for educational aerospace competitions, demonstrating the growing adoption of modular embedded systems within experimental rocketry environments. In [6], an Arduino-based rocket flight computer for a small model rocket is presented, where an Arduino Nano, BMP180 barometric sensor, MPU6050 inertial sensor, and SD-card data logging were used to collect flight data and compare the measured launch response with OpenRocket simulations. However, that work was mainly focused on low-cost avionics and offline post-flight data analysis, while the present study extends the scope to the complete design, fabrication, and experimental testing of a reusable 3D-printed rocket platform with PETG structural components, Raspberry Pi-based onboard sensing, wireless telemetry, camera integration, and repeated flight validation. Although existing studies demonstrate substantial progress in simulation-based design and embedded avionics, several limitations remain. Current low-cost platforms often provide limited integration between additive manufacturing, onboard sensing, telemetry, and experimental validation. Furthermore, simulation tools such as OpenRocket remain sensitive to uncertainties associated with aerodynamic drag, environmental disturbances, and propulsion variability. Consequently, there remains a need for reusable and accessible experimental platforms capable of combining additive manufacturing, embedded sensing, and flight-data acquisition within a unified framework suitable for educational and research-oriented aerospace applications.

### 1.3. Objectives and Contributions

**Figure 1:**
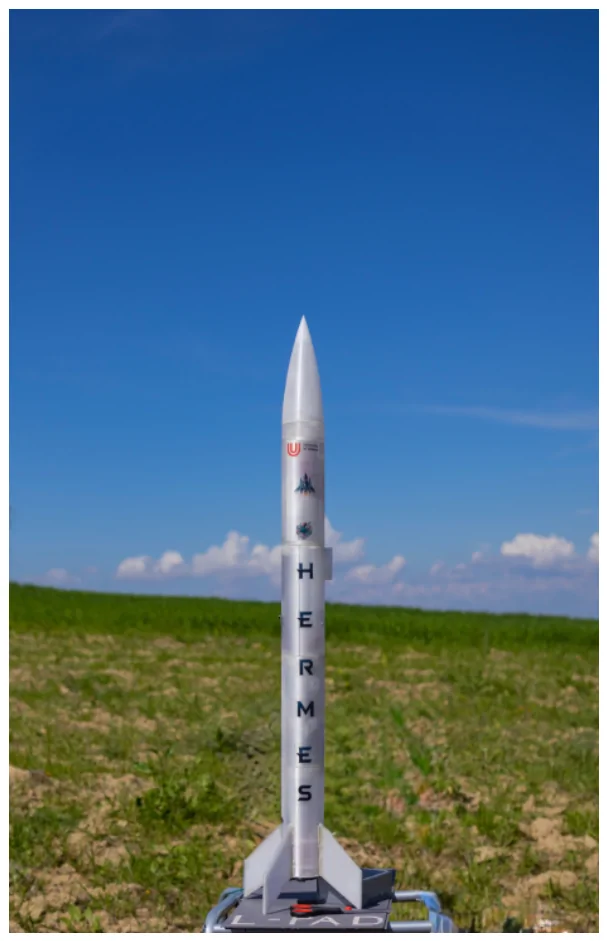
Hermes rocket prior to the first flight campaign.

The Hermes Rocket Project aimed to design, fabricate, and experimentally evaluate a reusable 3D-printed model rocket integrating onboard sensing, telemetry, and flight-data acquisition capabilities. The system was developed around a custom flight computer based on the Raspberry Pi Zero 2W platform (Raspberry Pi Ltd, Cambridge, UK) and incorporated an inertial measurement unit, barometric pressure sensor, GPS module, and wireless telemetry architecture. The assembled Hermes rocket is shown in Figure 1. The project additionally sought to evaluate the agreement between simulated and experimentally measured flight performance through repeated flight testing.

The primary contributions of this study are summarized as follows:

- Development of a reusable 3D-printed rocket platform manufactured using PETG components and modular design principles.
- Design and implementation of a low-cost flight computer integrating onboard sensing, telemetry, and real-time flight-data acquisition capabilities.
- Experimental validation of the integrated platform through four flight campaigns, including comparison between OpenRocket simulations and measured flight data.
- Assessment of practical implementation challenges associated with telemetry reliability, GPS acquisition, recovery-system performance, and environmental effects during flight operations.
- Demonstration of the feasibility of combining additive manufacturing and commercially available embedded electronics for educational and experimental aerospace applications.

The main contribution of this work is not any single subsystem in isolation, but the integration of additive manufacturing, embedded sensing, wireless telemetry, and repeated experimental flight validation within a single reusable, open-source platform, together with the resulting experimental dataset comparing measured and simulated flight performance.

Compared with existing low-cost model-rocketry platforms, Hermes combines a set of capabilities that are not jointly present in prior work, as summarized in Table 1. Rohini et al. [2] characterized a custom solid propellant on a thrust stand but did not include any onboard sensing, telemetry, or instrumented flight data. Telaak and De Backer [4] presented a design-only flight computer without reporting flight test data, while Binz et al. [5] describe the CATS Vega flight computer, a mature open-source system offering higher sampling rates and substantially longer telemetry range than the present work, representing a more capable commercial-grade benchmark. Our own prior work [6] used a barometer and IMU with SD-card logging but no wireless telemetry, GPS, or camera. The Hermes platform extends this progression by combining a 9-axis IMU, barometric sensor, GPS module, and onboard camera with real-time telemetry and repeated (four-flight) experimental verification, in a way none of the cited systems do on its own.

### 1.4. Paper Organization

The remainder of this paper is organized as follows. Section 2 presents the theoretical background on model rocket flight dynamics, additive manufacturing, and embedded avionics. Section 3 presents the system design methodology, including the structural configuration, material selection, and additive manufacturing process. Section 4 describes the flight computer architecture, onboard sensors, and telemetry system. Section 5 discusses the propulsion system and motor integration. Section 6 presents the aerodynamic modeling and stability analysis performed using OpenRocket. Section 7 describes the recovery system and parachute deployment mechanism. Section 8 presents the experimental setup and pre-flight validation procedures. Section 9 analyzes the experimental flight results and compares the measured data with simulation predictions. Finally, Section 10 summarizes the main findings, discusses system limitations, and provides recommendations for future work.

## 2. Theoretical Background

### 2.1. Model Rocket Flight Dynamics

**Figure 2:**
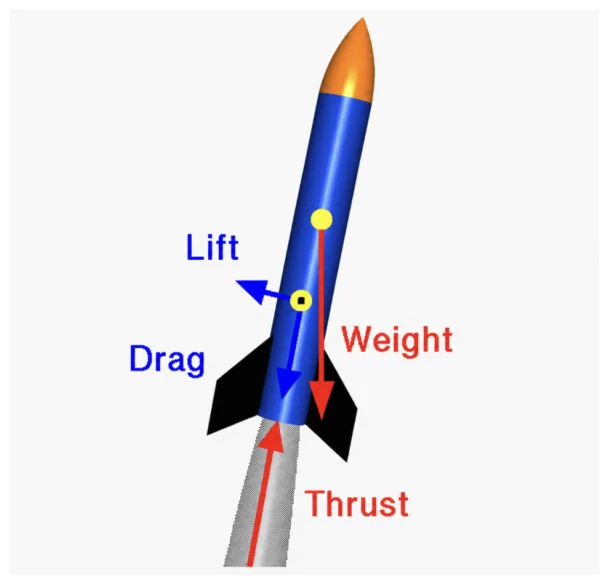
Primary aerodynamic forces acting on a model rocket during flight Adapted from [9].

Model rockets operate under the combined influence of thrust, gravity, and aerodynamic forces during ascent and descent. The primary aerodynamic forces acting on the vehicle consist of thrust, drag, lift, and weight (Figure 2), which collectively determine, which collectively determine flight stability and trajectory behavior [1]. Stable flight conditions require the center of pressure (CP) to remain behind the center of gravity (CG), thereby generating a restoring aerodynamic moment in response to external disturbances.

The translational motion of the rocket can be described using Newton’s second law:

(1) $$F_{\mathrm{net}}=ma=F_{\mathrm{thrust}}-F_{\mathrm{drag}}-F_{\mathrm{gravity}}$$

where *m* represents the rocket mass and *a* denotes acceleration.

The thrust force generated by the propulsion system is expressed as

(2) $$F_{\mathrm{thrust}}=\dot{m}v_{e}$$

where $\dot{m}$ is the propellant mass flow rate and $v_{e}$ is the exhaust velocity.

Aerodynamic drag is estimated using

(3) $$F_{\mathrm{drag}}=\frac{1}{2}C_{d}\rho Av^{2}$$

where $C_{d}$ is the drag coefficient, ρ is the air density, *A* is the reference cross-sectional area, and *v* is the rocket velocity.

The gravitational force acting on the rocket is given by

(4) $$F_{\mathrm{gravity}}=mg$$

where *g* denotes the gravitational acceleration.

The theoretical velocity increment achievable by a rocket system can be estimated using the Tsiolkovsky rocket equation [7]:

(5) $$\Delta v=v_{e}\ln\left(\frac{m_{0}}{m_{f}}\right)$$

where $m_{0}$ and $m_{f}$ represent the initial and final rocket masses, respectively.

The propulsion-system efficiency is commonly characterized through the specific impulse:

(6) $$I_{sp}=\frac{F}{\dot{m}g_{0}}$$

where $g_{0}$ is the standard gravitational acceleration.

Assuming negligible aerodynamic losses, the maximum altitude corresponding to a known vertical velocity may be approximated through conservation of mechanical energy [8]:

(7) $$h=\frac{v^{2}}{2g}$$

These analytical relations form the theoretical basis for the flight-performance assessment and simulation-validation procedures presented in this study.

### 2.2. Additive Manufacturing in Aerospace Applications

Additive manufacturing has become increasingly relevant in aerospace engineering due to its capability to produce lightweight and geometrically complex structures with reduced manufacturing cost and development time. Fused filament fabrication (FFF), in particular, has enabled rapid prototyping and iterative structural optimization using thermoplastic materials such as Polylactic Acid (PLA), Polyethylene Terephthalate Glycol (PETG), Acrylonitrile Butadiene Styrene (ABS), and carbon-fiber-reinforced polymers.

The aerospace industry has increasingly adopted additive manufacturing techniques for structural components, propulsion systems, and rapid prototyping applications. Organizations such as SpaceX, NASA, and Relativity Space have demonstrated the feasibility of large-scale additive manufacturing in aerospace production environments [10]. Within model rocketry applications, additive manufacturing provides substantial flexibility in controlling wall thickness, internal infill geometry, structural reinforcement, and aerodynamic shaping while significantly reducing fabrication complexity compared with conventional machining methods.

### 2.3. Embedded Flight Computers and Telemetry Systems

Modern model-rocketry platforms increasingly rely on embedded avionics systems for flight-data acquisition, telemetry transmission, and recovery-system control. Flight computers integrate onboard sensors, microcontrollers, wireless communication interfaces, and power-management subsystems within a compact architecture capable of operating under dynamic flight conditions.

Typical onboard sensing architectures include inertial measurement units for acceleration and orientation estimation, barometric sensors for altitude estimation, and GPS modules for trajectory tracking. Telemetry systems additionally enable real-time transmission of flight data to a ground station for monitoring and post-flight analysis.

Despite the increasing accessibility of embedded avionics platforms, several implementation challenges remain. Flight computers must maintain stable operation under vibration loading, rapid acceleration, pressure variation, and temperature fluctuations while simultaneously satisfying strict mass and power constraints. Furthermore, wireless telemetry reliability and sensor synchronization remain critical considerations in low-cost aerospace experimentation. These challenges motivate the development and experimental validation of integrated avionics architectures such as the system presented in the Hermes Rocket Project.

## 3. Rocket Design and Manufacturing

### 3.1. System Architecture and Conceptual Design

The Hermes rocket was designed as a reusable experimental platform integrating onboard avionics, telemetry, and flight-data acquisition capabilities within a lightweight modular structure. The primary design objectives included achieving stable flight performance, supporting repeated launch operations, and enabling real-time acquisition of sensor and trajectory data during flight. A target apogee of approximately 150 m was selected to satisfy experimental and safety requirements while remaining compatible with the selected propulsion system. The payload for this project consisted of the integrated avionics stack (flight computer, sensors, camera, and battery); no separate scientific or deployable payload was carried. The propulsion system was constrained to a total impulse within Class D. A minimum thrust-to-weight ratio of 5:1 at ignition was set as a design requirement to ensure reliable launch clearance and initial stability, consistent with standard model rocketry design practice.

The rocket architecture incorporated a custom flight computer, onboard camera, telemetry subsystem, power supply, and parachute recovery mechanism. OpenRocket was used for preliminary aerodynamic design, trajectory prediction, and stability analysis, while Autodesk Fusion 360 was employed for detailed computer-aided design (CAD) modeling and structural integration.

**Figure 3:**
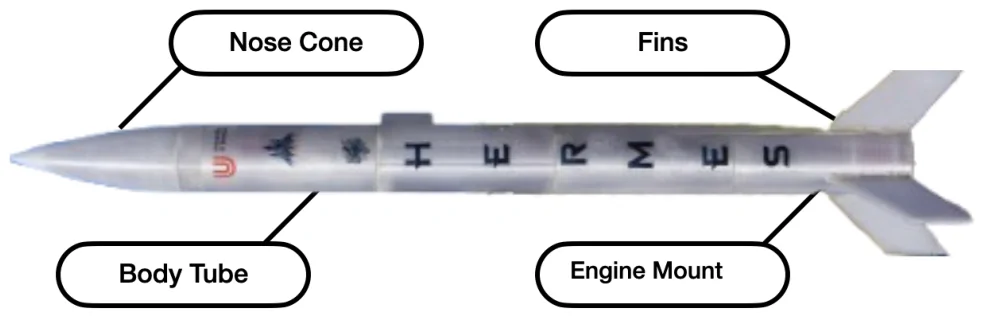
Hermes rocket with labeled structural components.

The main structural components of the rocket are illustrated in Figure 3. The body tube served as the primary load-bearing structure and housed the avionics system, parachute, and propulsion interfaces. An ogive nose cone geometry was selected to reduce aerodynamic drag while maintaining manufacturability through fused filament fabrication. Four rear fins were integrated to provide passive aerodynamic stabilization during ascent, while the aft section incorporated a clustered motor-mount assembly for the propulsion system.

### 3.2. Material Selection

Material selection represented a critical design consideration due to its influence on structural integrity, thermal resistance, manufacturability, and total vehicle mass. Several commercially available fused filament fabrication materials commonly used in aerospace prototyping applications were evaluated, including PLA, PETG, ABS, and carbon-fiber-reinforced nylon [10].

Although carbon-fiber-reinforced nylon exhibited the most favorable mechanical properties (Table 2), PETG was selected as the optimal compromise between structural performance, thermal resistance, manufacturing reliability, and cost efficiency [11,12]. Compared with PLA, PETG additionally provided improved ductility and reduced brittleness under dynamic loading conditions associated with launch and landing operations. Notably, PETG’s glass transition temperature (∼80 to 85  °C) exceeds the maximum avionics-bay temperatures recorded during ejection across all flights (approximately 34 to 50  °C, Section 9.2), indicating the structural components did not approach thermal softening during any ejection event, and confirming PETG’s suitability for the thermal loads encountered in this application.

### 3.3. Structural Design

The rocket geometry was primarily constrained by the avionics-system dimensions and the propulsion configuration. The body tube outer diameter was fixed at 67 mm to accommodate the flight computer and sensor modules while maintaining compatibility with the selected propulsion architecture.

The main body tube was designed with a length of 670 mm and a wall thickness of 1.8 mm. The resulting internal radius was calculated as

(8) $$r_{i}=33.5-1.8=31.7\;\mathrm{mm}$$

**Figure 4:**
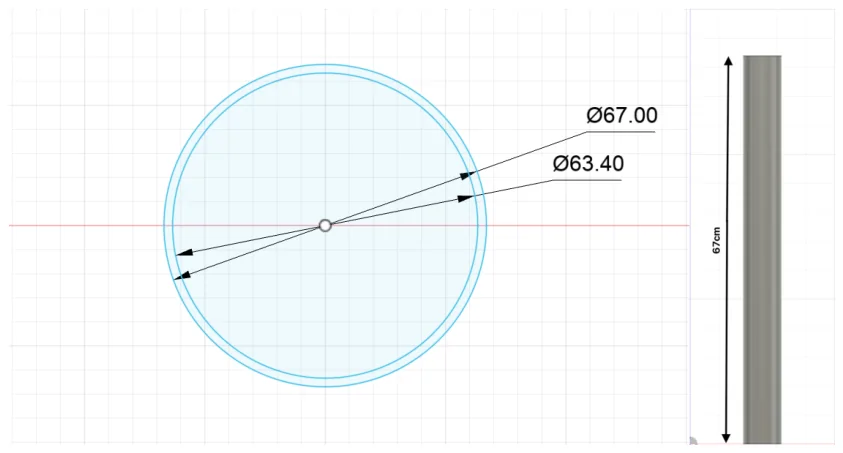
CAD model of the Hermes body tube developed in Fusion 360.

The corresponding structural volume was approximately $247.02\;{\mathrm{cm}}^{3}$, resulting in an estimated PETG mass of approximately 284.08g assuming a material density of $1.15\;g/{\mathrm{cm}}^{3}$ (Figure 4).

**Figure 5:**
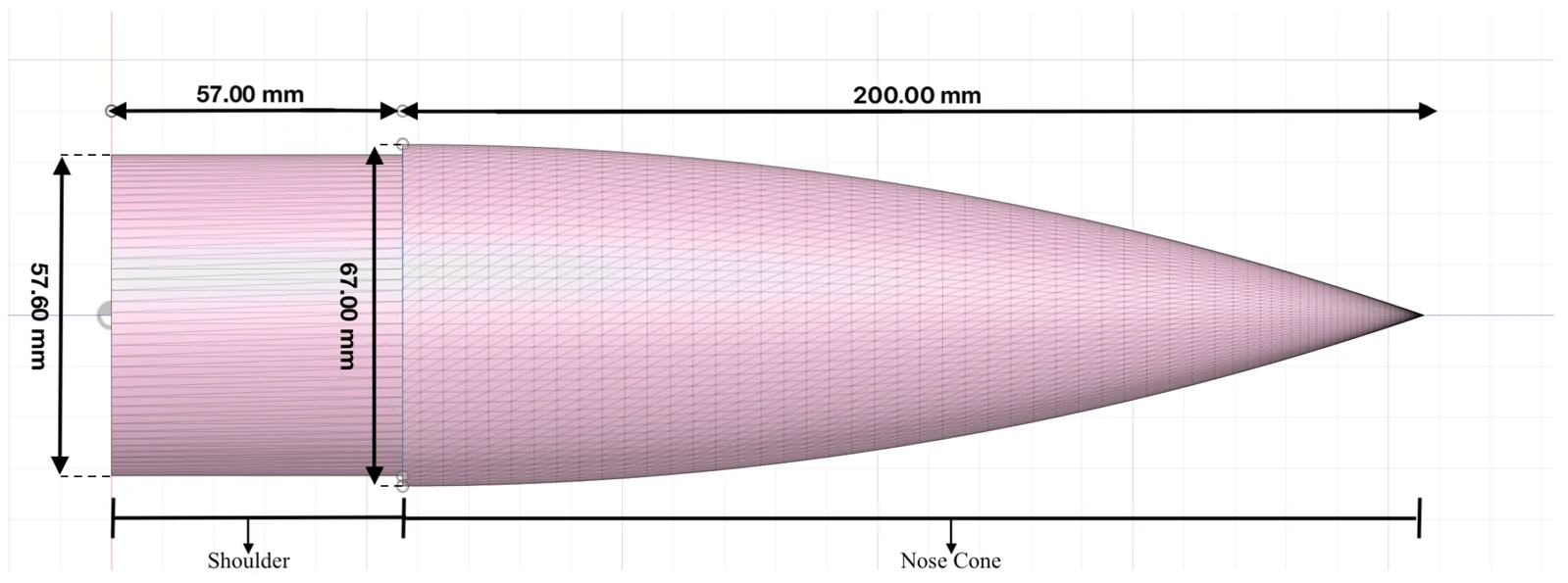
CAD model of the Hermes nose cone and shoulder assembly.

An ogive nose cone geometry was selected due to its favorable aerodynamic characteristics and manufacturing simplicity. The nose cone length was fixed at 200 mm, while the integrated shoulder interface measured 57 mm to ensure secure attachment to the forward body tube and support parachute deployment. The combined nose-cone assembly mass was approximately 69.2g (Figure 5).

**Figure 6:**
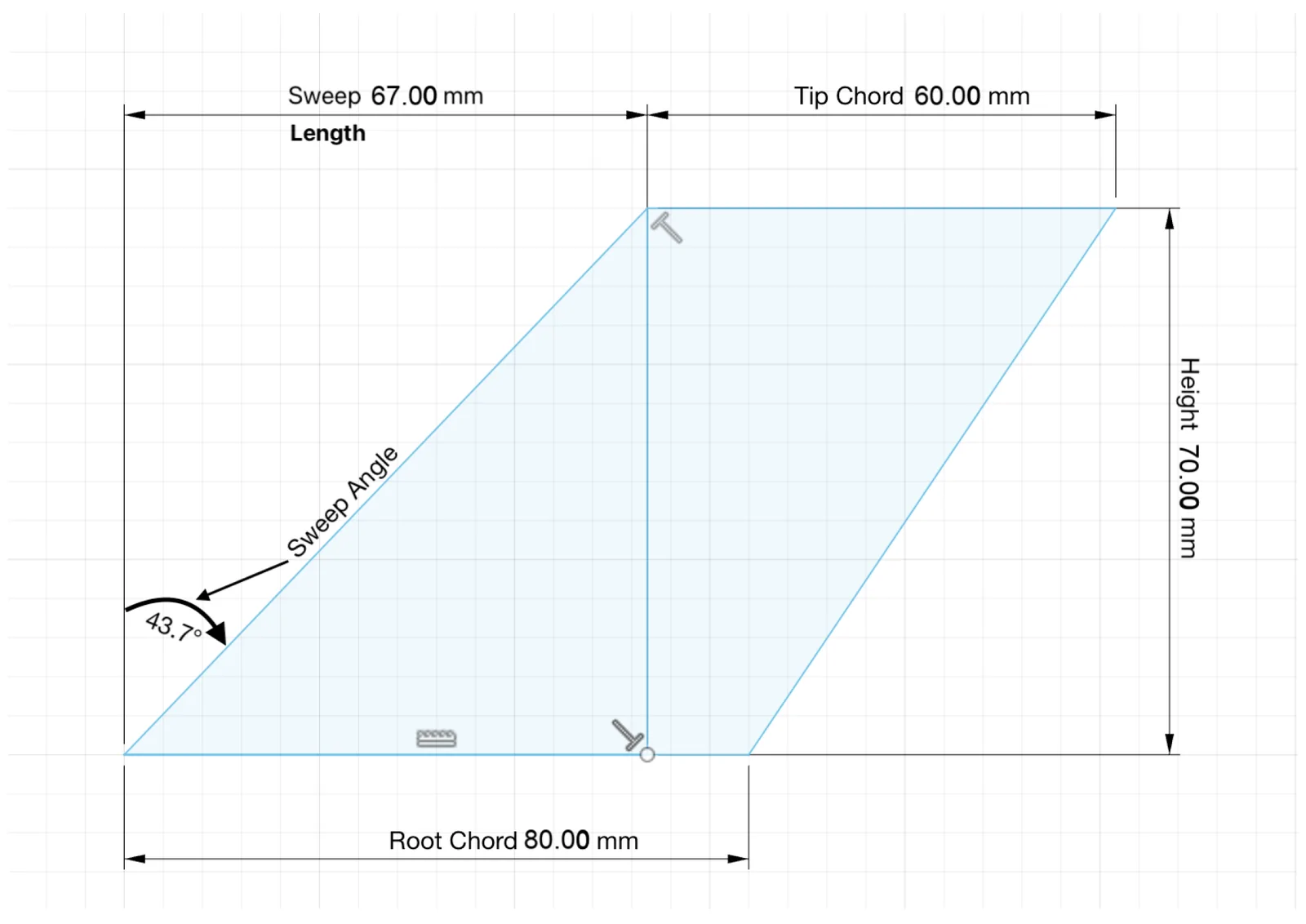
Hermes fin geometry modeled in Fusion 360.

**Figure 7:**
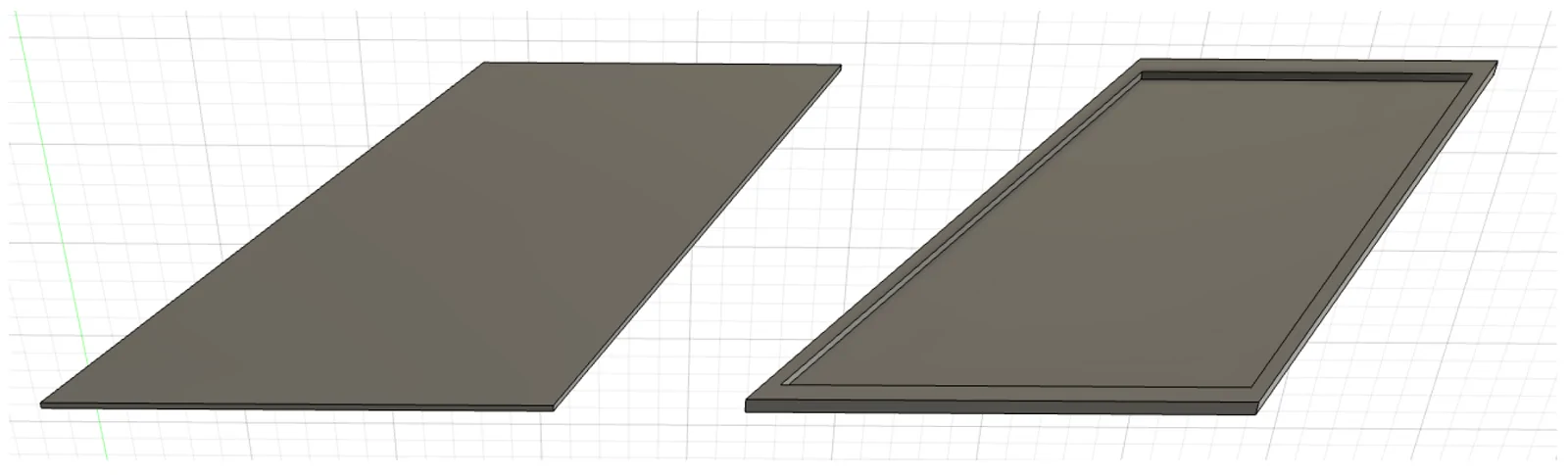
Internal hollow fin structure used for mass reduction.

A four-fin configuration was selected to improve passive aerodynamic stability during ascent. The fins were designed with hollow internal structures to reduce mass while preserving structural rigidity. Compared with a solid configuration, the hollow design reduced the fin mass by approximately 10 g per fin [1]. Each fin featured a height of 70 mm, root chord of 80 mm, tip chord of 60 mm, and thickness of 5 mm, resulting in a total fin mass of approximately 74.5g (Figure 6), with the hollow internal structure shown in Figure 7.

### 3.4. Additive Manufacturing Process

**Figure 8:**
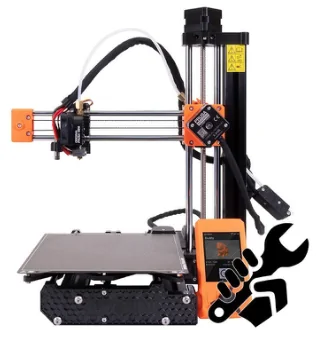
Original Prusa MINI+ fused filament fabrication printer used for manufacturing.

All structural components were manufactured using an Original Prusa MINI+ fused filament fabrication printer (Figure 8) compatible with PETG filament. The printer provided layer heights ranging from 0.05 mm to 0.25 mm and a maximum build volume of 170×170×170mm.

Due to the build-volume constraints, the rocket body was manufactured as multiple modular sections subsequently assembled into the final structure. The nose cone and body sections were designed to ensure repeatable alignment and simplified maintenance access while maintaining adequate structural rigidity during flight operations.

### 3.5. Assembly and Structural Integration

Permanent structural joints were implemented using J-B Weld Plastic Bonder epoxy adhesive, particularly for fin attachment and nose-cone assembly. To support modularity and internal maintenance access, detachable interfaces were additionally incorporated between body-tube sections.

Two modular joining approaches were evaluated: screw-based ring adapters and threaded cylindrical interfaces. The final configuration employed a threaded M58 × 1.5 ISO metric interface designed in Fusion 360, providing improved accessibility to internal avionics components while reducing assembly complexity during pre-flight preparation and post-flight maintenance.

## 4. Embedded Avionics and Sensor Integration

### 4.1. Avionics System Architecture

The Hermes rocket incorporated an onboard embedded avionics system responsible for flight-data acquisition, telemetry transmission, sensor synchronization, and post-flight data logging. The avionics architecture was designed to operate under the mass, power, and geometric constraints imposed by the rocket structure while maintaining sufficient computational capability for real-time sensor processing.

**Figure 9:**
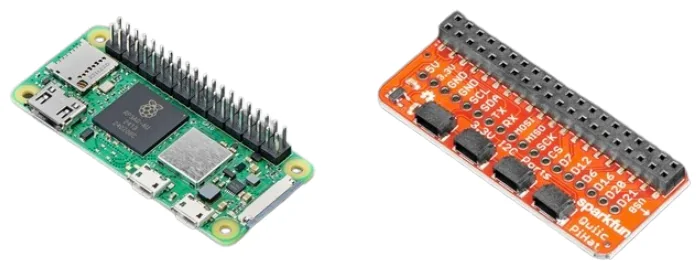
Raspberry Pi Zero 2W and SparkFun Qwiic/STEMMA QT interface board. Image reproduced with permission of Adafruit Industries [13].

The integrated avionics system consisted of a Raspberry Pi Zero 2W microcontroller platform, inertial and environmental sensors, a GPS module, an onboard camera system, and a lightweight battery subsystem. Sensor interconnections were implemented through a SparkFun Qwiic/STEMMA QT HAT (SparkFun Electronics, Niwot, CO, USA) (Figure 9) utilizing the Inter-Integrated Circuit (I^2^C) communication protocol, thereby reducing wiring complexity and improving modularity.

The primary functions of the avionics system included:

- Real-time acquisition of pressure, temperature, inertial, and positioning data;
- Telemetry transmission to a ground station during flight;
- Local onboard storage of experimental flight data;
- Video recording for post-flight trajectory analysis.

In this manuscript, ’telemetry’ refers specifically to the real-time wireless transmission of flight data to the ground station, as distinct from onboard data logging to the microSD card, which is described separately in Section 4.5.

### 4.2. Hardware Selection and Sensor Configuration

The Raspberry Pi Zero 2W was selected as the primary onboard processing platform due to its favorable balance between computational performance, compact dimensions, wireless communication capability, and power consumption. Alternative microcontroller platforms, including the Arduino Nano, Arduino Due, and STM32 Blue Pill, were evaluated but were ultimately considered unsuitable due to limitations associated with sensor integration, processing capability, or camera compatibility. The Raspberry Pi Zero 2W provided a quad-core processor and 512 MB RAM, enabling simultaneous sensor acquisition, telemetry transmission, and onboard video recording [13].

**Figure 10:**
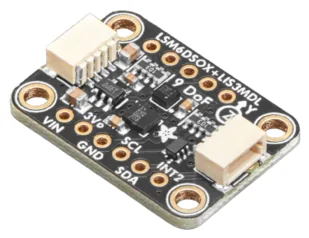
Adafruit DPS310 digital pressure sensor. Image reproduced with permission of Adafruit Industries [13].

Altitude and environmental measurements were acquired using the Adafruit DPS310 barometric pressure sensor, selected due to its low power consumption, compact dimensions, and high-resolution pressure measurements with an estimated altitude resolution of approximately 20 mm [13] (Figure 10).

**Figure 11:**
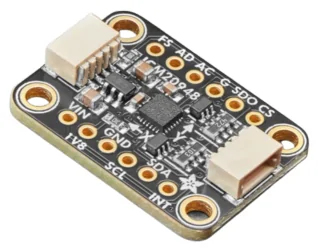
Adafruit TDK InvenSense ICM-20948 inertial measurement unit. Image reproduced with permission of Adafruit Industries [13].

Attitude and acceleration measurements were obtained using the Adafruit TDK InvenSense ICM-20948 inertial measurement unit. Compared with lower-cost six-axis alternatives such as the MPU6050, the selected sensor additionally incorporated a three-axis magnetometer, enabling full nine-axis orientation estimation. The ICM-20948 also provided improved power efficiency and reduced physical dimensions, making it more suitable for compact embedded aerospace applications [13] (Figure 11).

**Figure 12:**
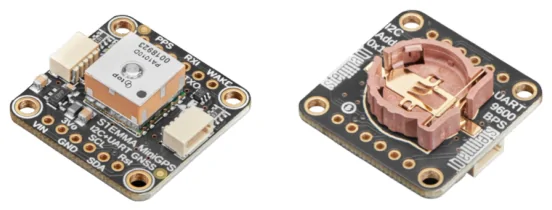
Adafruit Mini GPS PA1010D module. Image reproduced with permission of Adafruit Industries [13].

A GPS module (Adafruit Mini GPS PA1010D, Adafruit Industries, New York, NY, USA) (Figure 12) was integrated into the avionics system for trajectory tracking and positioning; however, this subsystem did not achieve satellite lock during ground testing or flight operations, as discussed in detail in Section 4.4.

**Figure 13:**
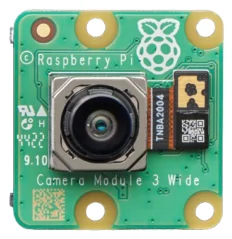
Raspberry Pi Camera Module 3 with wide-angle lens. Image reproduced with permission of Adafruit Industries [13].

Visual flight recording was implemented using the Raspberry Pi Camera Module 3 equipped with a $120^{\circ }$ wide-angle lens. The camera system provided 12-megapixel imaging capability, autofocus functionality, and high dynamic range performance for post-flight trajectory assessment (Figure 13).

### 4.3. Power System Design

The avionics power subsystem was designed to satisfy the operational requirements of the onboard electronics while minimizing additional structural mass. The combined current consumption of the Raspberry Pi, sensors, GPS module, and camera system was estimated at approximately 465mA.

Assuming a continuous operating duration of 15 min, the required battery capacity was estimated as

(9) C=I×t=465mA×0.25h=116.25mAh

A Cellevia LP903450 lithium-polymer battery rated at 3.7V and 800mAh was selected to provide substantial operational margin while additionally contributing to mass balancing within the rocket structure. A voltage step-up converter was integrated to satisfy the operating voltage requirements of the Raspberry Pi platform.

### 4.4. Sensor Placement and Mechanical Integration

Sensor placement within the rocket was selected to maintain mass balance and minimize disturbances to the vehicle center of gravity. The heaviest components, namely the battery and Raspberry Pi, were positioned approximately 170–175 mm from the base of the body tube to reduce pitch instability during ascent.

The camera module (approximately 3 g) required precise counterbalancing to maintain the vehicle’s center-of-gravity within the design envelope. A GPS module (Adafruit Mini GPS PA1010D, approximately 3 g) was integrated at a corresponding axial location to serve as a mass-balance counterweight. Removing it would have required reprinting a counterbalance mass or repositioning other components to preserve CG, so it was retained purely as ballast for all four flights.

The inertial measurement unit and barometric sensor were mounted approximately 10 cm above the central avionics section, while the GPS and camera modules were positioned approximately 6 cm above the inertial sensing assembly. The camera system required an external opening within the body tube to enable unobstructed visual recording during flight.

A major implementation challenge involved protecting the onboard electronics from the propulsion-system ejection charge while simultaneously preserving sufficient airflow for parachute deployment. To address this issue, a dedicated protective structure consisting of two semi-circular printed barriers was integrated into the lower avionics compartment to shield the embedded electronics from combustion byproducts and thermal exposure.

### 4.5. Telemetry and Ground-Station Architecture

The telemetry architecture was designed to support both real-time flight monitoring and post-flight experimental analysis. During flight operations, telemetry data were transmitted as plain-text User Datagram Protocol (UDP) datagrams over a mobile Wi-Fi hotspot to a ground-station laptop running a dedicated Python (version 3.12.7) receiver script. The ground-station software utilized Matplotlib (version 3.10.1) for real-time and post-flight visualization of pressure, temperature, altitude, acceleration, angular velocity, orientation, and horizontal displacement measurements. The telemetry update rate matched the onboard sensor logging rate for each flight (1 Hz in Flight 1, up to 100 Hz in Flight 4, as described in Section 4.6). Although the wireless connection remained stable during initial ascent, signal loss occurred shortly after launch due to the limited range and environmental sensitivity of the selected Wi-Fi communication architecture.

To ensure complete data preservation independently of telemetry reliability, all sensor measurements were simultaneously logged to the Raspberry Pi microSD card throughout each flight campaign.

A dedicated ground-based range test was conducted prior to flight to characterize telemetry link performance. During the test, the flight computer was powered on while the operator walked progressively farther from the ground station, monitoring the live telemetry feed. In a representative test conducted on 20 March 2025, stable telemetry reception was maintained for approximately the first 60 m of separation before the connection to the mobile hotspot was lost. This measured range was substantially lower than the intended telemetry range of 200 m (Following disconnection, the onboard script continued to operate uninterrupted, and all sensor and video data were successfully recorded to the microSD card, confirming that local data logging was independent of telemetry link availability.

Several reproducibility-relevant aspects of the telemetry implementation remain limitations of the current system. Transmitted packets did not include sequence numbers or other identifiers to detect missing or out-of-order deliveries, consistent with the connectionless nature of UDP. No mechanism was implemented to detect, reorder, or retransmit lost packets, and packet loss during transmission was not quantitatively measured. Because telemetry values were generated from the same onboard acquisition loop used for local logging, telemetry and locally stored data shared a common timestamp source, although this synchronization was not independently verified. Future work will address these limitations through structured packet framing, sequence numbering, and quantitative link-quality assessment.

### 4.6. Data Processing and Measurement Methodology

The ICM-20948 IMU was operated using its default configurable ranges (accelerometer and gyroscope), consistent with the specifications listed in Section 4.2. Prior to first use, an initial calibration was performed to estimate accelerometer and gyroscope bias offsets. This calibration was not repeated before every flight, which is acknowledged as a limitation. Orientation, acceleration, and angular rate data are reported directly in the sensor’s body-fixed frame, without transformation to an external or world-referenced coordinate frame. Sensor synchronization was achieved by polling all sensors sequentially within a single acquisition loop on the Raspberry Pi, with sample timing determined by the loop’s operating frequency (1 Hz in Flight 1, 10 Hz in Flights 2–3, and 100 Hz in Flight 4). No independent hardware timestamping or inter-sensor clock synchronization was implemented. Barometric altitude was computed relative to a ground-level reference pressure recorded at the start of each flight: the first pressure sample logged after system activation on the launch pad was used as the local reference (zero-altitude) pressure, and all subsequent altitude values for that flight were calculated relative to this reference using the sensor library’s standard pressure-to-altitude conversion. This approach compensates for day-to-day variation in local atmospheric pressure but was applied independently for each flight, using only that flight’s own initial pressure reading as the reference. For Flight 4, the reported total acceleration was adjusted by subtracting a constant value of $9.81\;m/s^{2}$ from the measured magnitude to approximate net (gravity-compensated) acceleration, this correction was not applied in Flights 1 to 3. Orientation angles (pitch, roll and yaw) shown in the telemetry plots were derived directly from raw accelerometer and gyroscope readings. For data visualization purposes only, a smoothing filter was applied to the plotted time-series to improve readability; the underlying stored sensor data remains unfiltered. The sampling rate was increased progressively across the flight campaign (1 Hz, 10 Hz, and 100 Hz, respectively) as part of iterative system improvements. As discussed in Section 9.2.1, the initial 1 Hz rate was found insufficient to resolve short-duration transient events, meaning that peak values (e.g., peak acceleration, peak velocity) measured in early flights may underestimate the true peak magnitude relative to later, higher-rate flights. This limits direct quantitative comparison of transient peak metrics across the flight campaign, although overall trends in flight profile (altitude and general velocity behavior) remain comparable.

## 5. Propulsion System

### 5.1. Motor Selection and Performance Characteristics

The propulsion system was designed to provide sufficient thrust-to-weight ratio while remaining compatible with cost and availability constraints associated with student-developed experimental platforms. Given the total vehicle mass and the requirement to achieve a target apogee of approximately 150 m, a clustered solid-motor configuration was selected.

The Klima D9-5 solid rocket motor was chosen due to its availability, well-characterized thrust profile, and compatibility with OpenRocket simulation libraries [14]. Key motor specifications are summarized below:

- Total impulse: 20 N·s
- Average thrust: 9 N
- Peak thrust: 25 N
- Propellant mass: 16 g
- Burn duration: 2.1 s
- Ejection delay: 5 s

To satisfy the required thrust-to-weight ratio of 5:1 and ensure stable liftoff conditions, a three-motor cluster configuration was implemented, achieving a combined average thrust-to-weight ratio exceeding 5:1 at ignition.

### 5.2. Thrust Profile and Simulation Model

**Figure 14:**
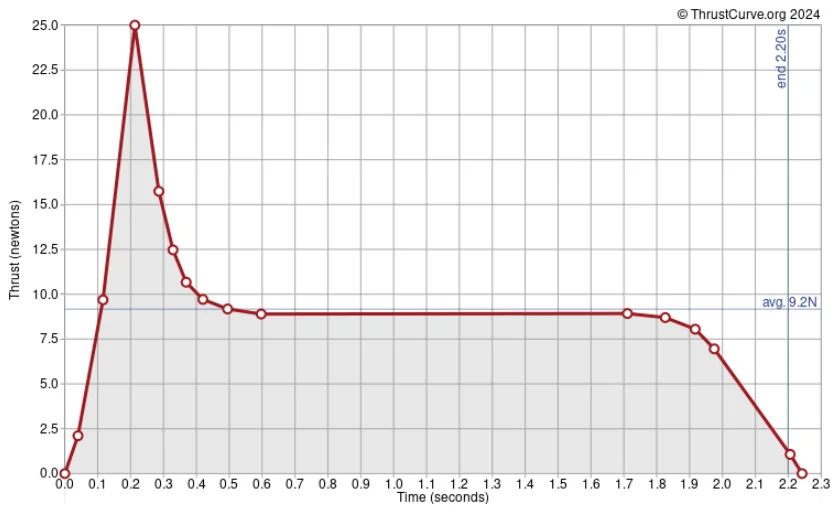
Thrust-time profile of the Klima D9-5 solid rocket motor [14].

The Klima D9-5 motor exhibits a transient thrust profile characterized by an initial peak followed by a decay toward a quasi-steady average thrust level. The peak thrust occurs shortly after ignition, reaching approximately 25 N, while the average thrust stabilizes at approximately 9.2 N over the burn duration of 2.1 s (Figure 14).

This time-dependent thrust behavior was incorporated into the OpenRocket simulation environment to improve the fidelity of predicted ascent dynamics and to enable comparison with experimentally recorded acceleration profiles. The relatively smooth thrust decay contributes to reduced structural loading transients compared with highly impulsive propulsion systems, although variability between nominal and actual motor performance remains a key source of uncertainty in flight prediction.

### 5.3. Motor Integration and Structural Configuration

The three-motor cluster was integrated into a custom-designed engine mount assembly developed in Autodesk Fusion 360. The mount consisted of a reinforced ring-based structure with an outer diameter of 63.2 mm and a wall thickness of 5 mm, designed to ensure axial alignment and structural rigidity during thrust generation.

The engine assembly was positioned at the aft section of the rocket and secured using a combination of mechanical constraints and epoxy bonding. Two structural support rings were installed at axial positions 1 cm and 5 cm from the base of the body tube to distribute load transfer and maintain alignment of the thrust vector with the vehicle’s longitudinal axis.

Proper alignment of the propulsion system was critical to minimizing thrust-induced torque and preventing off-axis loading, which could significantly affect stability and sensor measurements during the boost phase. The integration approach therefore directly influenced both the dynamic flight behavior and the quality of onboard inertial measurement data acquired during ascent.

The three motors were mounted parallel to the body tube’s central axis, ensuring that the combined thrust line remained coincident with the longitudinal axis along which the vehicle’s center of gravity is located, independent of axial CG shift due to propellant mass depletion during the burn.

## 6. Simulation and Stability Analysis

The OpenRocket model was configured with measured vehicle parameters to match the physical Hermes rocket as closely as possible. The vehicle total mass was set to 0.736 kg with propellant loaded and 0.655 kg dry (after motor burnout). The center of gravity was positioned at 570 mm from the nose tip, and the center of pressure (computed by OpenRocket using empirical aerodynamic methods) was located at 703 mm, yielding a static stability margin of 1.98 calibers, well above the minimum 1.0 Cal threshold for stable flight. The aerodynamic drag coefficient was computed by OpenRocket based on the rocket’s geometric profile, the parachute was modeled with a drag coefficient of 1.5, consistent with standard flat-sheet parachute assumptions. The launch rail was specified as 1.0 m in length, matching the physical launch pad. Wind speed inputs to the simulation were derived from measured atmospheric conditions recorded during each flight test, rather than using a single nominal wind value, to improve fidelity of the predicted trajectory. The ejection delay (time from motor burnout to parachute deployment) was set to 5 s, consistent with the actual motor specification and the deployment strategy described in Section 7.

### 6.1. Static Stability and Design Criteria

Static stability of the vehicle was evaluated to ensure predictable aerodynamic behavior during ascent and to maintain alignment between the vehicle axis and the velocity vector. Stability is governed primarily by the relative positions of the center of gravity (CG) and center of pressure (CP), where a stable configuration requires the CG to remain ahead of the CP along the longitudinal axis of the vehicle [1].

In this configuration, aerodynamic restoring moments are generated in response to angular perturbations, enabling the vehicle to naturally align with the freestream flow. Three stability regimes are typically identified: stable, neutral, and unstable flight conditions, corresponding respectively to CG located ahead of CP, coincident with CP, or behind CP.

For quantitative assessment, the stability margin was expressed in calibers (Cal), defined as

(10) $$\mathrm{Cal}=\frac{d_{\mathrm{CG}-\mathrm{CP}}}{D}$$

where $d_{\mathrm{CG}-\mathrm{CP}}$ is the axial distance between CG and CP and *D* is the body tube diameter. A stability margin between 1 and 2 Cal is generally considered appropriate for passive aerodynamic stability in model rocketry applications [1,15].

**Figure 15:**
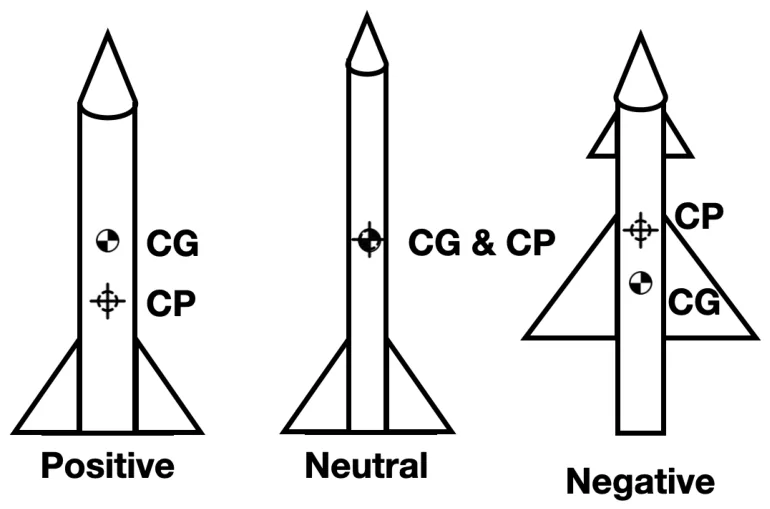
Static stability configuration illustrating CG and CP relationship.

For the Hermes configuration, OpenRocket analysis yielded CG and CP locations at 570 mm and 703 mm, respectively, corresponding to a stability margin of 1.98 Cal. This value indicates a stable configuration with sufficient margin to accommodate mass variations and minor aerodynamic uncertainties during flight (Figure 15).

### 6.2. Mass Distribution and Inertial Balancing

Mass distribution plays a critical role in determining both static stability and dynamic response during ascent. The vehicle was designed to ensure that high-mass subsystems, including the propulsion cluster and power system, were aligned with the longitudinal axis to minimize unwanted pitch or yaw moments.

**Figure 16:**
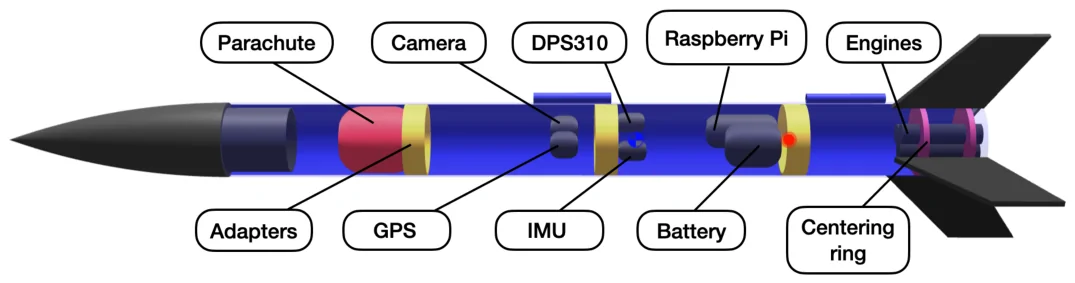
Mass distribution layout of onboard subsystems.

Electronic subsystems were arranged symmetrically where possible to reduce lateral imbalance. The propulsion assembly constituted the dominant mass contribution at the aft section, while avionics components were distributed along the central body tube to maintain CG alignment throughout the mission profile (Figure 16).

This configuration was implemented to minimize inertial asymmetries that could influence both aerodynamic stability and sensor measurements, particularly during the boost phase where accelerations are highest.

### 6.3. Numerical Simulation Framework

Flight dynamics were modeled using the OpenRocket simulation environment, which implements a combination of empirical aerodynamic models and numerical integration techniques for trajectory prediction [15]. The aerodynamic stability model is based on Barrowman equations for CP estimation, while drag forces are computed using the decomposed drag coefficient formulation given in Equation (3), where the total $C_{d}$ aggregates contributions from body, fin, base, and parasitic drag components.

The translational motion of the rocket is solved numerically using a fourth-order Runge–Kutta integration scheme:

(11) $$\frac{d^{2}x}{dt^{2}}=\frac{F_{\mathrm{thrust}}-F_{\mathrm{drag}}-F_{\mathrm{gravity}}}{m}$$

with time-dependent thrust profiles imported from manufacturer motor data [14].

### 6.4. Model Assumptions and Sources of Uncertainty

Despite its widespread use in model rocketry design, the simulation framework relies on several simplifying assumptions that introduce deviations between predicted and experimental results. These include rigid-body assumptions, axisymmetric geometry, and steady aerodynamic flow conditions.

Additional assumptions include the use of nominal thrust curves independent of environmental variation, and standard atmospheric models that do not account for localized wind fields or turbulence. These simplifications are particularly relevant in low-altitude flight regimes where transient effects and manufacturing tolerances can significantly influence trajectory behavior. A summary of key modeling assumptions and their implications is provided in Table 3.

These limitations motivate the experimental validation approach adopted in this study, where onboard sensor measurements are used to quantify deviations between modeled and observed flight behavior.

## 7. Recovery System Design

### 7.1. Descent Dynamics and Recovery Requirements

The recovery system was designed to ensure safe vehicle descent, structural reusability, and preservation of onboard sensor integrity following apogee. In addition to structural considerations, the recovery phase is critical for maintaining the continuity and usability of recorded flight data, particularly for post-flight validation of onboard inertial and barometric measurements. A passive parachute-based recovery system was implemented to achieve a controlled terminal descent velocity in the range of 4–6 m/s, which is commonly considered suitable for minimizing impact loads on lightweight experimental aerospace platforms.

### 7.2. Parachute Sizing and Aerodynamic Model

The parachute diameter was determined based on the equilibrium condition between gravitational force and aerodynamic drag at terminal velocity. Under steady descent conditions, the force balance can be expressed as

(12) $$mg=\frac{1}{2}\rho v_{t}^{2}C_{d}A$$

where *m* is the total vehicle mass, *g* is gravitational acceleration, ρ is air density, $v_{t}$ is terminal velocity, $C_{d}$ is the drag coefficient, and *A* is the projected parachute area.

Assuming a hemispherical parachute geometry with an effective drag coefficient of approximately 1.5 and standard atmospheric density of 1.225 kg/m^3^, the resulting parachute diameter was determined to be approximately 0.77 m for the target descent velocity range.

### 7.3. Structural Design and Deployment Mechanism

The parachute canopy was constructed from a lightweight, low-porosity fabric selected to ensure adequate drag generation with low added mass. An octagonal semi-spherical geometry was adopted to simplify fabrication while maintaining near-axisymmetric aerodynamic performance.

**Figure 17:**
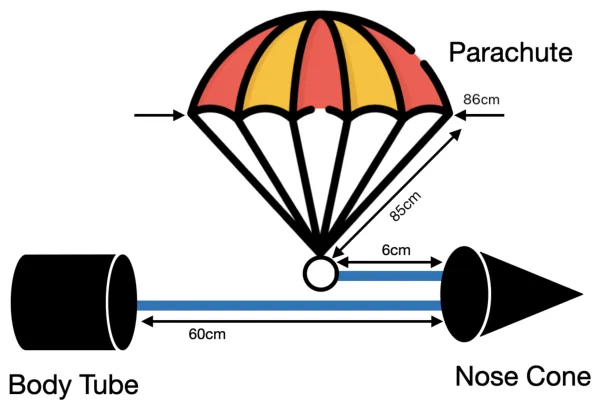
Schematic of the parachute recovery system architecture, showing key component lengths and connection points.

Load transfer during descent was managed using an eight-line suspension system to distribute loads evenly across the canopy. The shock cord system consisted of two elastomeric segments: a primary 60 cm section connecting the nose cone to the body tube, and a secondary 6 cm section linking the parachute assembly to a swivel joint to reduce torsional loading during descent (Figure 17).

Deployment was triggered using a pyrotechnic ejection charge integrated within the propulsion system, activated after a 5 s motor delay following burnout. The delay was chosen to approximate the simulated time-to-apogee, so that parachute deployment would occur close to the point of zero vertical velocity.

### 7.4. Safety and Launch Procedures

All flight tests were conducted on private land with the explicit permission of the landowner. Basic safety precautions were observed during each launch, including maintaining a safe standoff distance from the launch pad during ignition and ascent, and keeping a fire extinguisher on site throughout each test campaign. Launches were only conducted under favorable weather conditions, with a self-imposed limit of wind speeds below 15 km/h and no launches attempted during rain or other adverse conditions. The intended landing and recovery zone coincided with the launch site itself, an open area selected in advance to provide sufficient clearance from the launch pad, personnel, and any surrounding structures. In the case of the Flight 2 recovery-system failure (Section 9.1), the uncontrolled descent occurred within this cleared area at a safe distance from all personnel, and no injury or property damage resulted.

## 8. Testing and System Verification

A series of ground and pre-flight tests were conducted to verify the functionality of the integrated avionics, recovery, power, and structural subsystems. The verification process focused on confirming correct functional operation of the onboard sensing system under conditions representative of flight operations. The corresponding system requirements and verification methods are summarized in Table 4.

### 8.1. Avionics and Data Integrity Verification (R-1 to R-3)

The embedded avionics system was evaluated to verify continuous sensor acquisition, onboard data storage, and camera operation under conditions representative of flight deployment. The system was powered and operated continuously while progressively increasing the distance from the ground station, causing wireless connectivity to be lost due to range limitations, in order to evaluate data persistence under signal loss conditions.

The onboard system maintained continuous sensor logging at 1 Hz without data interruption, and all recorded video data were successfully stored locally on the microSD storage medium. These results confirmed that the data acquisition pipeline is independent of telemetry link availability, ensuring full dataset preservation even in the event of in-flight communication loss. The initial sampling-rate requirement of 1 Hz (Flight 1) was found insufficient to resolve short-duration transient events such as peak acceleration and peak velocity; therefore, the requirement was subsequently revised to 100 Hz, which was implemented in Flight 4 (Section 9.2.4).

### 8.2. Recovery System Functional Testing (R-4)

The parachute deployment mechanism was verified through ground-based simulation of the ejection event. Compressed airflow was used to emulate the internal pressure release generated by the motor ejection charge. The system demonstrated successful separation of the nose cone and deployment of the recovery system in a single ground-based ejection test, confirming functional operation of the deployment mechanism.

### 8.3. Power System Endurance Testing (R-5)

The integrated avionics system was subjected to continuous operation testing to evaluate battery endurance under full system load, including sensor acquisition, GPS operation, and video recording. The system operated continuously for more than 25 min without instability or power interruption, exceeding the 15 min minimum operational requirement by approximately 10 min (a margin of roughly 1.7×). This margin substantially exceeds the actual flight durations observed across all four test flights, which ranged from approximately 30 to 40 s from launch to landing (Section 9) and provides sufficient endurance under the nominal flight conditions encountered during testing, including wind speeds of 7 to 15 km/h and ambient temperatures of 15 to 22 °C (Table 5).

### 8.4. Static Stability Verification (R-6)

Static stability was verified both analytically (Section 6.1, OpenRocket stability margin of 1.98 Cal) and experimentally, using a physical swing test prior to flight operations. The test confirmed that the vehicle’s center of gravity remained forward of the center of pressure, consistent with simulation results. This configuration indicated stable passive aerodynamic behavior during ascent, with sufficient stability margin to accommodate small variations in component placement and mounting tolerances (e.g., battery seating, wiring routing) between assembly instances, which could otherwise shift the initial CG position and reduce the static margin at liftoff.

## 9. Results and Discussion

Four flight tests of the Hermes model rocket were conducted to evaluate system performance, sensor reliability, and the agreement between experimental results and OpenRocket simulations. Each flight was instrumented with an onboard telemetry system recording temperature, barometric altitude, linear acceleration, angular velocity, orientation, and time-stamped flight events. Environmental conditions, including launch site elevation, ambient temperature, atmospheric pressure, humidity, and wind speed (Table 5), were incorporated into pre-flight simulations to improve model fidelity.

A key limitation across all flights was the GPS receiver’s failure to achieve satellite lock, both during ground-based pre-flight testing and in flight. The root cause was not identified. Candidate explanations such as cold-start acquisition time, antenna placement and orientation, shielding from surrounding avionics or the PETG structure, and limited ground-test duration due to battery conservation, were not individually tested or isolated, and are noted here only as plausible directions for future investigation rather than established causes. Consequently, no usable GPS data were obtained, and trajectory estimation relied solely on barometric altitude measurements and inertial sensor integration, without independent horizontal position reconstruction.

### 9.1. System Iteration and Flight-to-Flight Improvements

Progressive modifications between successive flights addressed three faults identified in Table 6: ejection-charge thermal exposure (Flight 1), premature data-logging termination due to unintended power-switch activation (Flight 2), and nose cone/shock cord separation at apogee (Flight 2). Each modification is described below. Despite these improvements, measured apogees remained consistently below simulated predictions, indicating persistent aerodynamic and propulsion-modeling limitations.

### 9.2. Flight Data Analysis

This section presents the analysis of onboard telemetry data acquired during each flight. Key performance indicators include vertical velocity, barometric altitude, and total acceleration, which correspond to the primary observable outputs available in OpenRocket simulations.

#### 9.2.1. Flight 1

Flight 1 (5 April 2025) was conducted with a sampling frequency of 1 Hz, which was later identified as insufficient to resolve peak transient events. Temperature increased from approximately 42.5 °C to 50 °C during the ejection event, indicating partial thermal penetration into the avionics bay.

**Figure 18:**
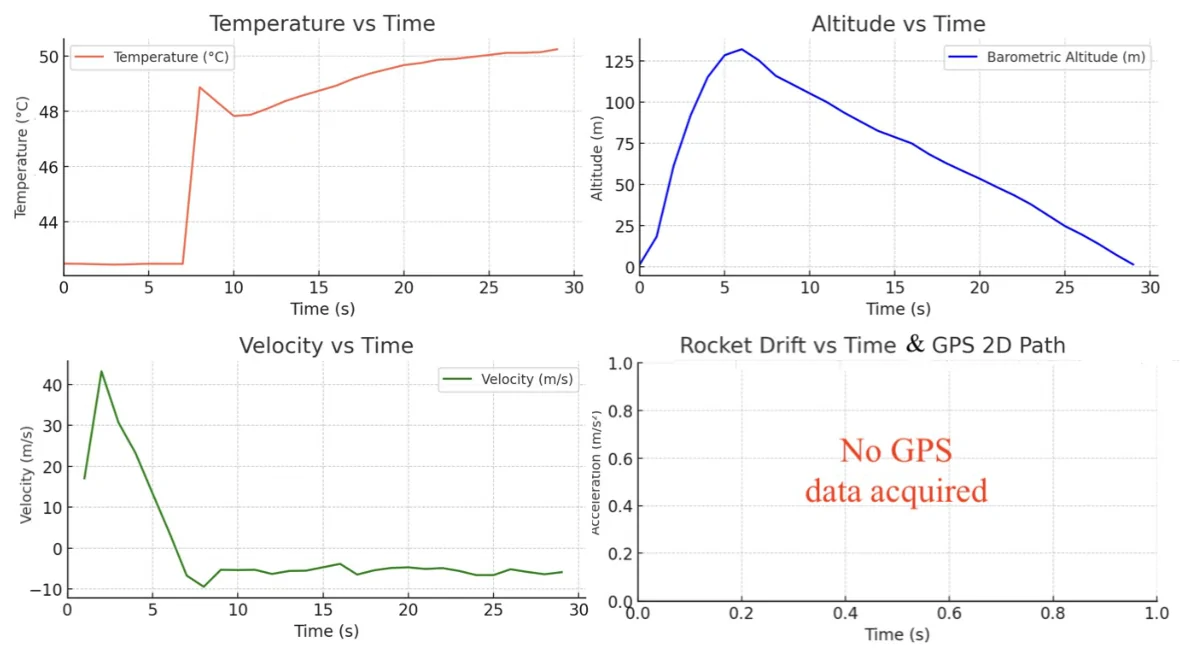
Flight 1 telemetry: temperature, altitude, and velocity.

**Figure 19:**
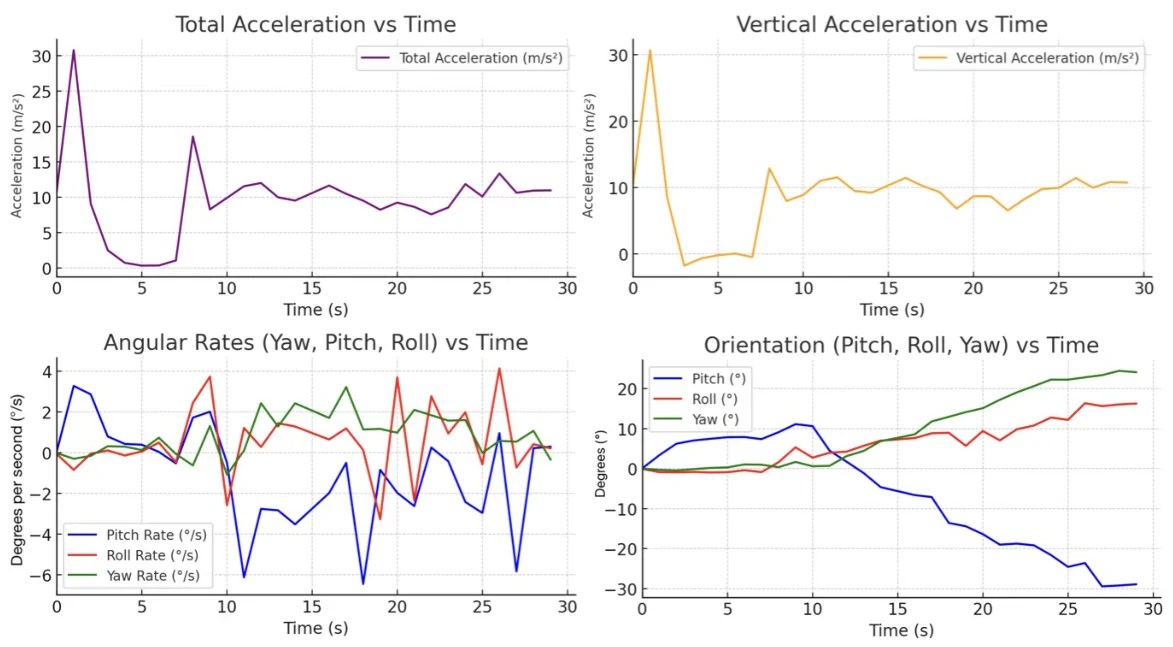
Flight 1 telemetry: acceleration, angular rates, and orientation.

The measured peak vertical velocity was approximately 42 m/s at 2 s, followed by parachute deployment and descent. Impact occurred at approximately 30 s. Attitude data indicated transient instability in pitch and roll during ascent, while yaw remained comparatively stable. After approximately 12 s, angular rates converged toward steady-state descent behavior as seen in Figure 18 and Figure 19.

**Figure 20:**
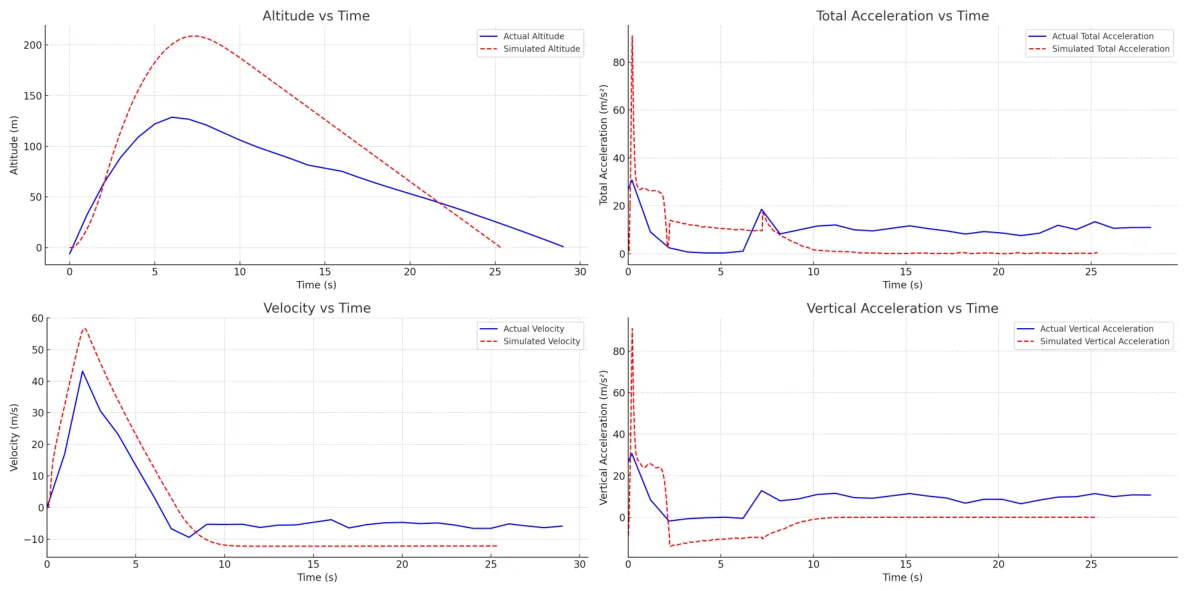
Flight 1: Comparison of measured and simulated telemetry.

Measured performance deviated from simulation predictions (Figure 20):

- Velocity: Peak measured velocity (42 m/s) was lower than the simulated value (55 m/s). Post-deployment descent velocity stabilized near −10 m/s, compared to −12.5 m/s in simulation.
- Acceleration: Measured peak acceleration reached 22 m/s^2^, with increased high-frequency fluctuations relative to simulation, likely due to aerodynamic disturbances and wind loading.
- Altitude: Measured apogee was significantly lower than simulated predictions.

#### 9.2.2. Flight 2

Flight 2 (18 April 2025) implemented a 10 Hz sampling rate and improved thermal protection for the avionics bay. Temperature variation during the ejection event was reduced to approximately 0.15 °C, compared to 8 °C in Flight 1.

Data acquisition terminated at approximately 9 s due to an unintended activation of the onboard power switch. Prior to termination, altitude increased consistently to approximately 130 m. A structural failure occurred near apogee, resulting in premature separation of the nose cone and uncontrolled descent.

**Figure 21:**
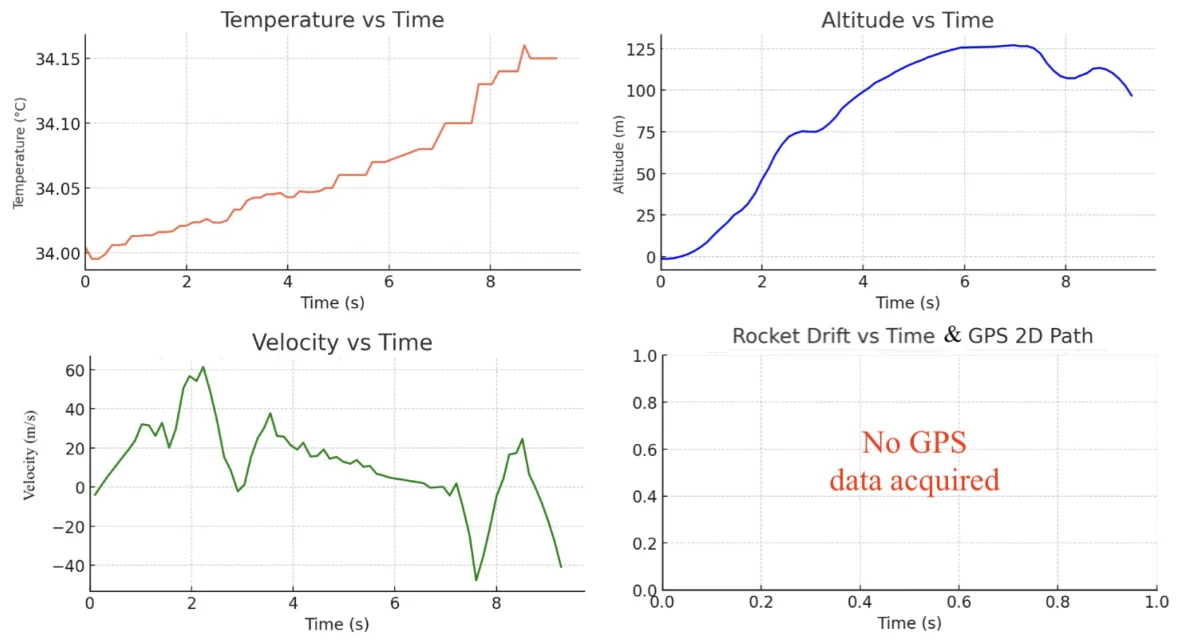
Flight 2 telemetry: temperature, altitude, and velocity.

**Figure 22:**
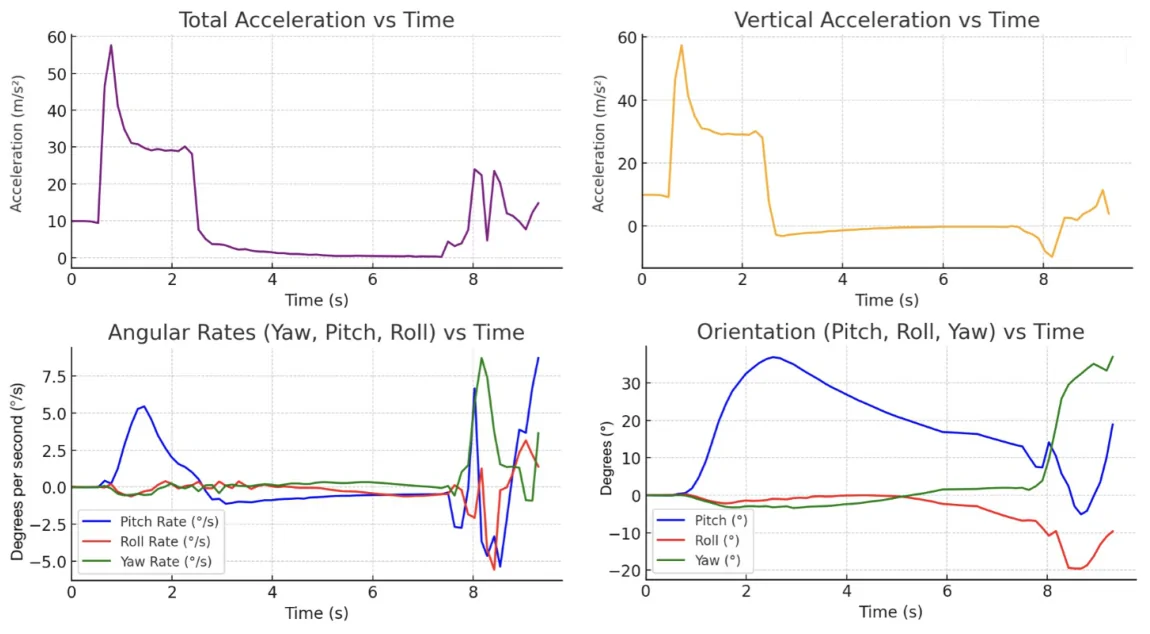
Flight 2 telemetry: acceleration, angular rates, and orientation.

Peak vertical velocity reached 55–60 m/s, followed by a high-speed descent phase without parachute stabilization. Maximum measured acceleration was approximately 60 m/s^2^, lower than simulated predictions (Figure 21 and Figure 22).

**Figure 23:**
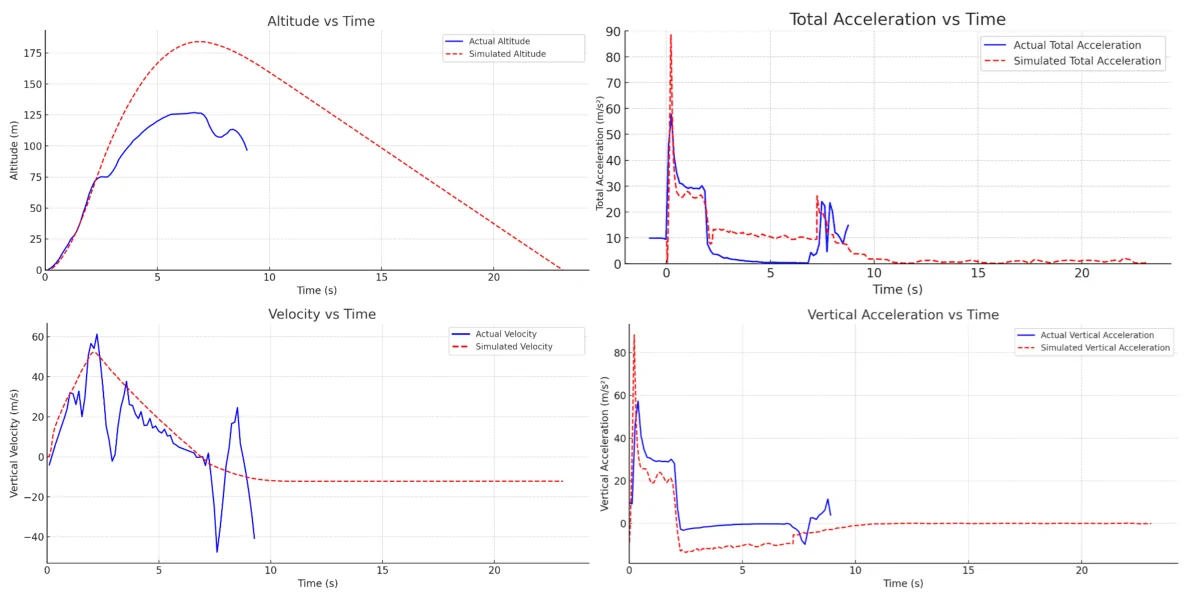
Flight 2: Measured vs. simulated telemetry.

Measured performance deviated from simulation predictions (Figure 23):

- Altitude: Maximum measured altitude was approximately 130 m prior to data loss.
- Velocity: Good agreement during powered ascent; divergence occurred during descent due to failure event.
- Acceleration: Lower than simulated peak values, attributed to thrust variability and unmodeled losses.

#### 9.2.3. Flight 3

**Figure 24:**
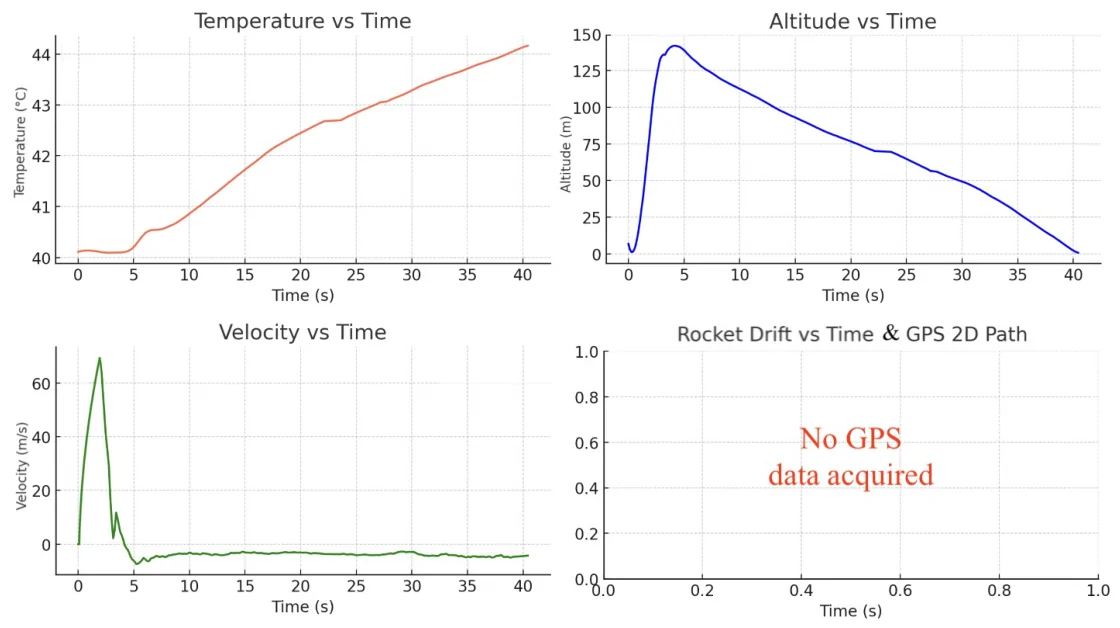
Flight 3 telemetry: temperature, altitude, and velocity.

Flight 3 (4 May 2025) incorporated structural reinforcement, removal of the mechanical power switch, and improved recovery system integration. Temperature increased from 40 °C to 44 °C during the ejection event, confirming improved thermal protection (Figure 24).

**Figure 25:**
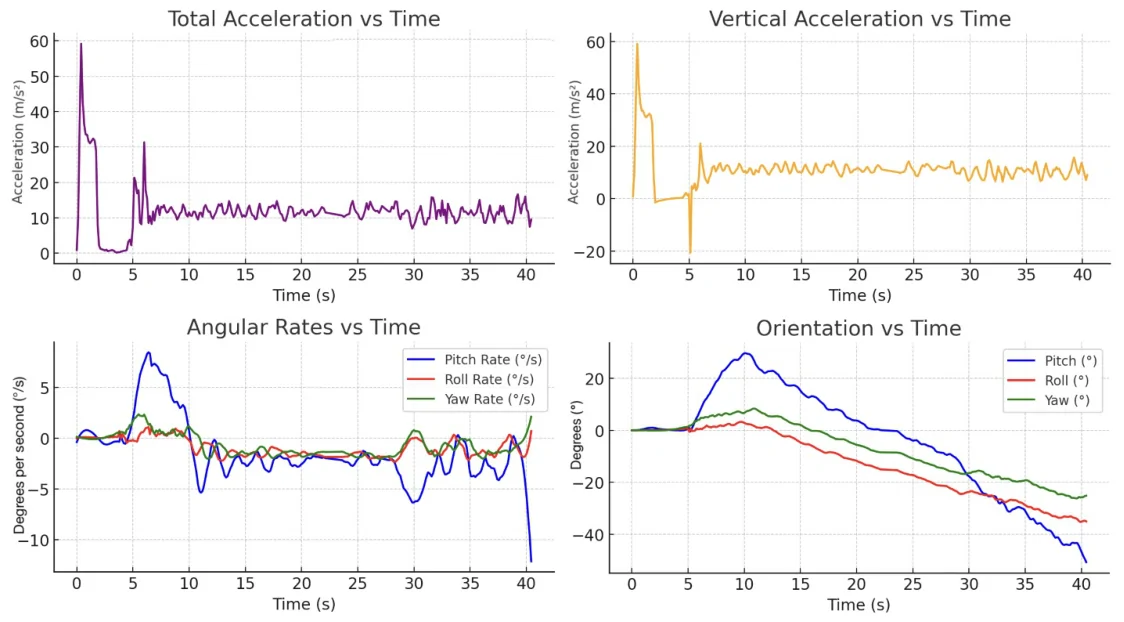
Flight 3 telemetry: acceleration, angular rates, and orientation.

**Figure 26:**
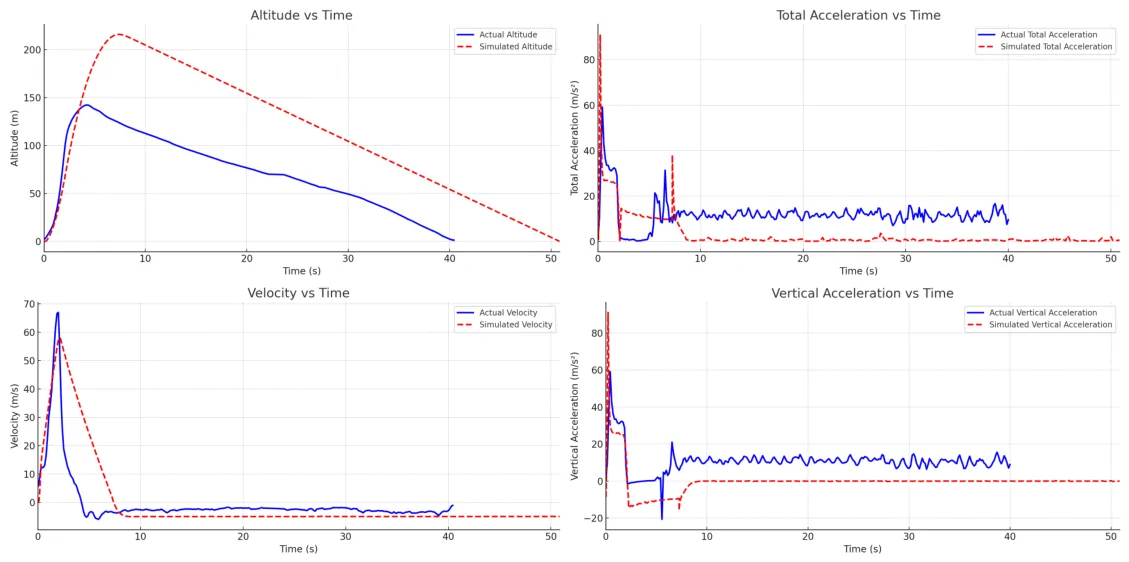
Flight 3: Measured vs. simulated telemetry.

Maximum measured altitude reached 143 m, compared to a simulated value of 210 m. Peak velocity exceeded 60 m/s, with earlier-than-predicted deceleration indicating increased aerodynamic drag or reduced effective thrust duration, as shown in Figure 24. The measured acceleration, angular rates, and orientation data also indicate a transient pitch deviation of approximately 20° near apogee, affecting ascent efficiency (Figure 25). Figure 26 compares the measured and simulated flight profiles, highlighting the lower-than-predicted apogee and earlier velocity decay.

The key observations from Flight 3 are summarized below:

- Altitude: 143 m (measured) vs. 210 m (simulated)
- Velocity: Peak exceeded 60 m/s, with earlier decay than predicted
- Stability: Observable pitch excursion prior to apogee

#### 9.2.4. Flight 4

**Figure 27:**
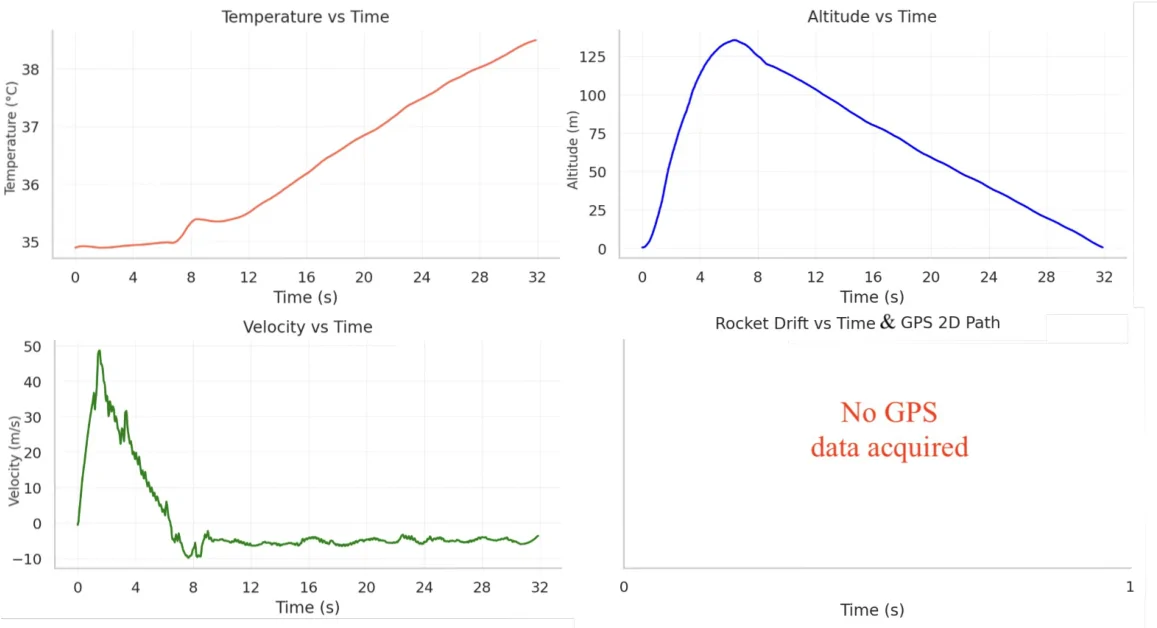
Flight 4 telemetry: temperature, altitude, and velocity.

**Figure 28:**
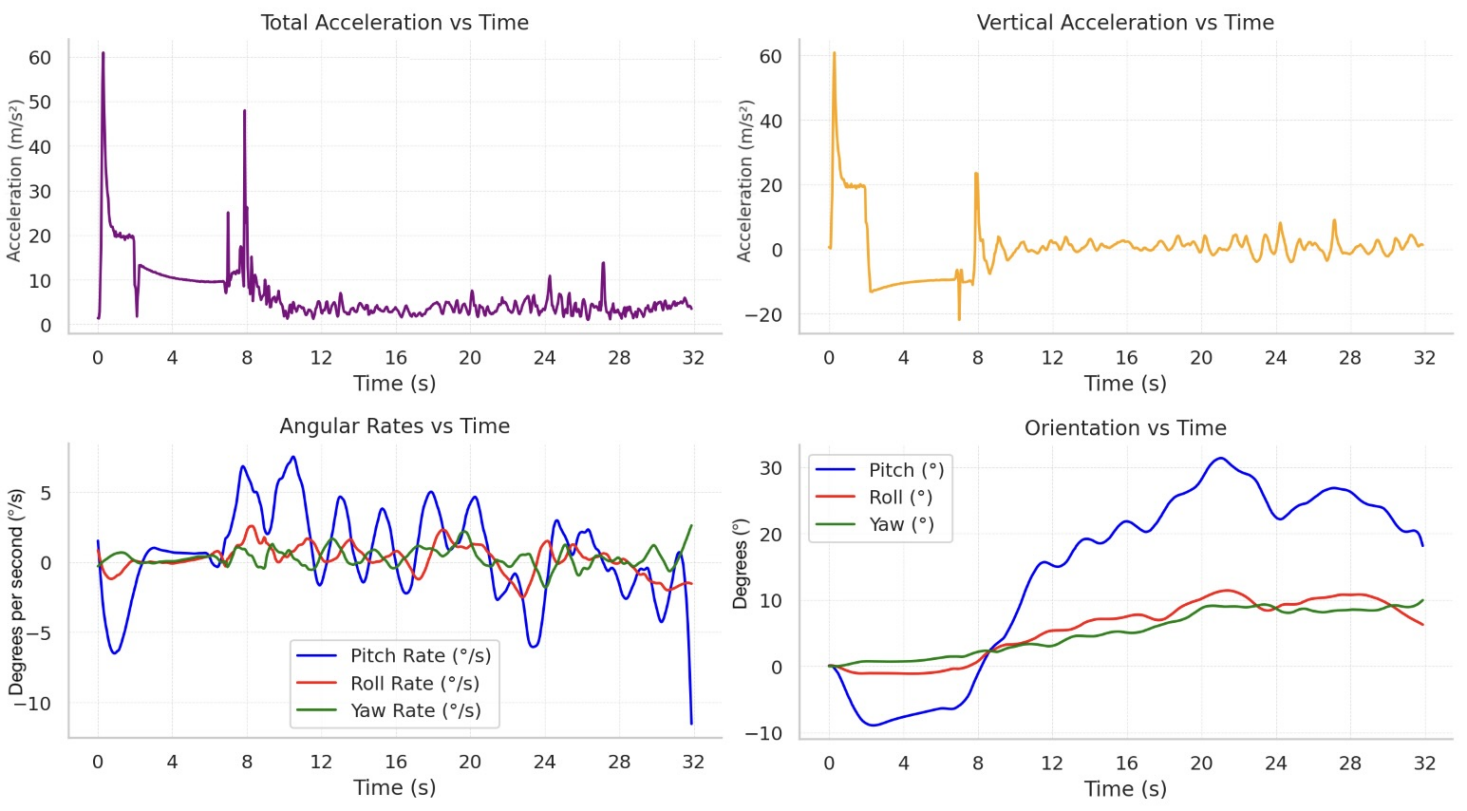
Flight 4 telemetry: acceleration, angular rates, and orientation.

Flight 4 (11 May 2025) implemented the final system configuration, including 100 Hz telemetry sampling, gravity-compensated acceleration processing, and improved fin alignment (Figure 27 and Figure 28).

The rocket reached a maximum altitude of 135 m. Peak velocity was approximately 50 m/s. Post-deployment descent velocity (−5 m/s) was lower in magnitude than simulated values, indicating higher-than-modeled parachute drag efficiency.

**Figure 29:**
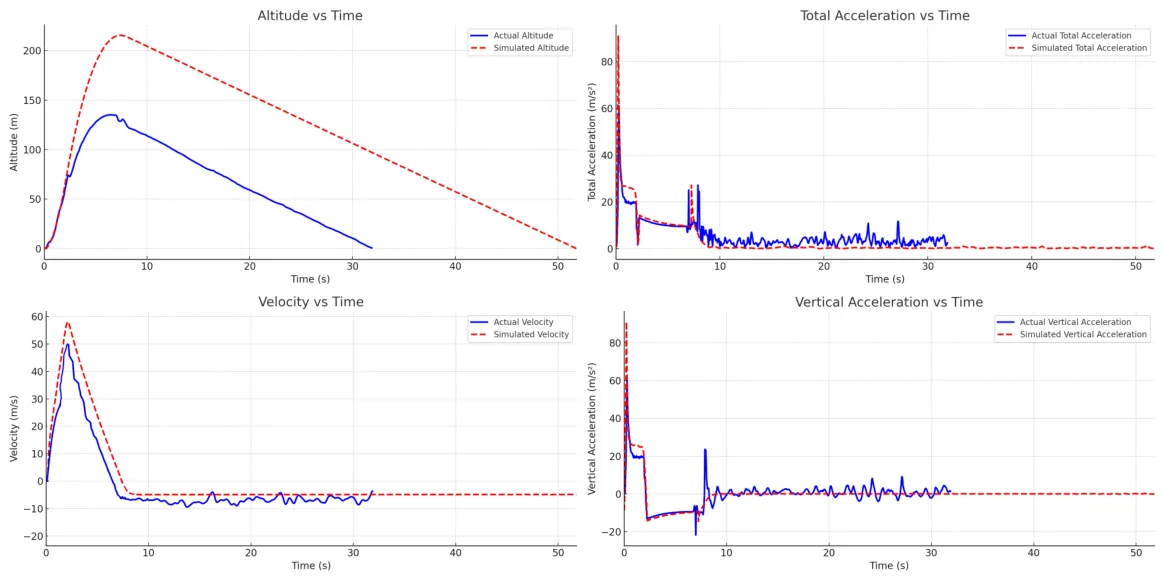
Flight 4: Measured vs. simulated telemetry.

Peak acceleration remained approximately 60 m/s^2^, consistent with previous flights, with improved temporal resolution confirming transient peak behavior (Figure 29).

- Altitude: 135 m
- Velocity: 50 m/s peak ascent velocity
- Acceleration: 60 m/s^2^ peak (gravity-compensated analysis)

### 9.3. Cross-Flight Performance Comparison

Across all flights, consistent performance trends were observed. Temperature control improved significantly after Flight 1 due to enhanced thermal shielding. Structural resilience of the PETG airframe was confirmed, including survival of an impact event from approximately 100 m altitude during Flight 2.

**Figure 30:**
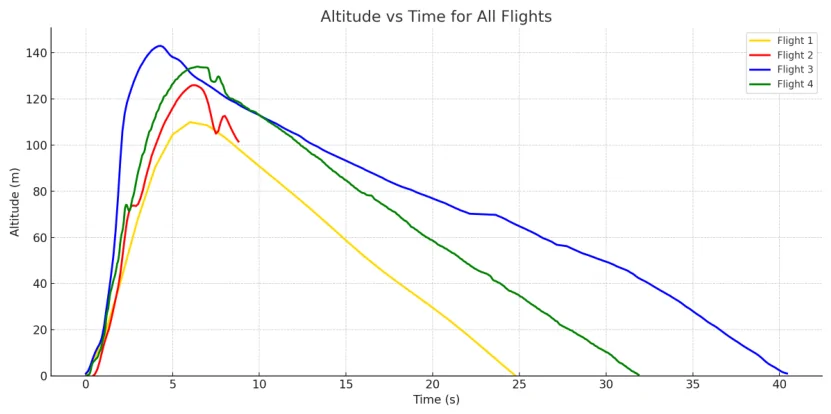
Altitude comparison across all flights.

**Figure 31:**
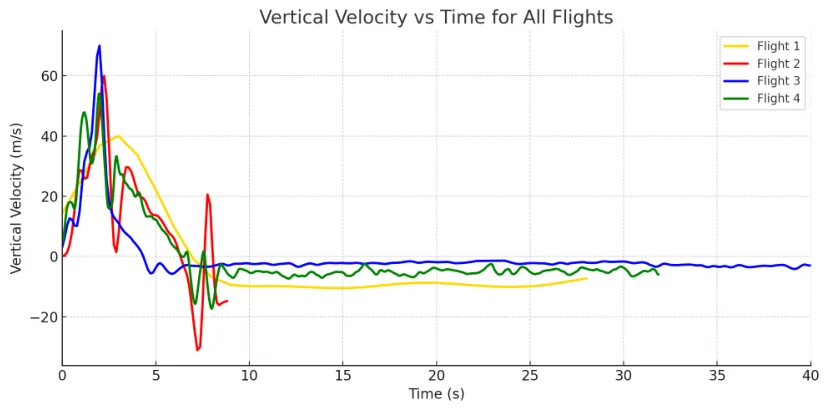
Vertical velocity comparison across all flights.

Measured apogee values remained consistently below simulation predictions, with maximum observed altitude of 143 m compared to simulated values exceeding 200 m Figure 30. Maximum vertical velocity stabilized near 50 m/s across all flights, indicating a repeatable propulsion-limited regime (Figure 31).

### 9.4. Discussion of Simulation Deviations

Simulation-to-experiment discrepancies were primarily attributed to: (i) inaccuracies in parachute modeling parameters, (ii) idealized motor thrust curves, (iii) unmodeled aerodynamic drag effects, and (iv) transient attitude instabilities during ascent.

**Figure 32:**
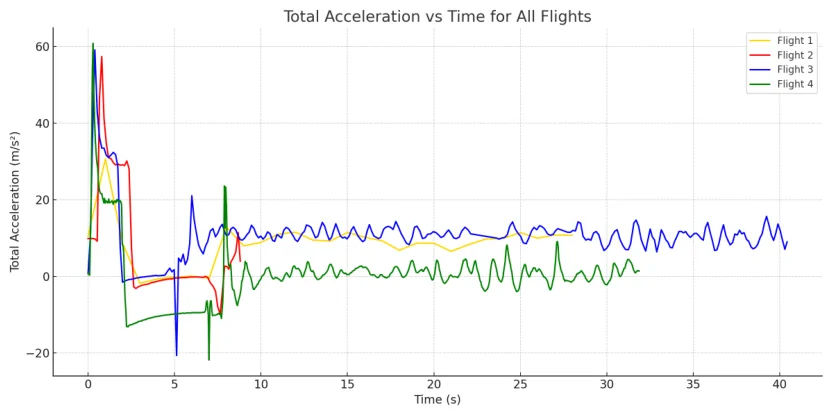
Total acceleration comparison across all flights.

Peak acceleration values (60 m/s^2^ measured vs. 90 m/s^2^ simulated) indicate systematic overestimation in the simulation model (Figure 32). Similarly, flight duration was consistently shorter than predicted due to reduced achieved apogee.

### 9.5. Flight Performance Model

A simplified physics-based Python model was developed as a first-order diagnostic tool to investigate how individual modeling assumptions affect predicted flight performance. The model divides flight into powered and ballistic phases, assuming constant thrust during motor burn and a constant drag coefficient throughout. Rather than providing an independently accurate trajectory prediction, the model is used to isolate the relative contributions of unmodeled drag and thrust variability to the discrepancies observed between OpenRocket simulations and measured flight data for altitude, velocity, and time-to-apogee.

The effective acceleration during powered ascent is defined as

$$a_{\mathrm{burn}}=\frac{T}{m}-g$$

where *T* is thrust, *m* is mass, and *g* is gravitational acceleration.

Model inputs correspond to Flight 4 conditions (Table 5). The model systematically overestimated performance due to neglect of time-varying drag and thrust fluctuations.

- Maximum altitude: 221.6 m (predicted) vs. 135 m (measured)
- Maximum velocity: 56.44 m/s (predicted) vs. 50 m/s (measured)
- Time to apogee: 7.85 s (predicted) vs. 6.57 s (measured)

While Table 7 presents both the simplified Python model and OpenRocket predictions for context, Table 8 focuses specifically on the OpenRocket comparison, as it represents the higher-fidelity simulation tool used for the primary design and validation process.

It should be noted that the four flights do not constitute repeated trials of a single fixed configuration. Each flight incorporated design or software modifications in response to issues identified in the preceding flight (Table 6), including changes to thermal protection, sampling rate, power circuitry, and fin alignment. Flights 1–3 are therefore presented as design-iteration and troubleshooting evidence, demonstrating the resolution of specific hardware and software faults. Flight 4 is representing the final system configuration, used as the primary basis for the quantitative comparison against simulation, as summarized in Table 8.

### 9.6. Overall Performance Assessment

The results confirm that while simplified analytical models and OpenRocket simulations provide useful first-order estimates, they systematically overpredict performance when compared with high-frequency telemetry data. The ideal velocity increment was estimated using the Tsiolkovsky rocket equation. For the three-motor cluster configuration, the combined propellant mass was 3 × 16 g = 48 g. Using a specific impulse of 127.5 s, the effective exhaust velocity was calculated as $v_{e}=I_{sp}\cdot g_{0}=127.5\times 9.81=1250.8$ m/s, yielding

$$\Delta v=v_{e}\ln\left(\frac{m_{0}}{m_{f}}\right)=1250.8\cdot \ln\left(\frac{m_{0}}{m_{f}}\right)=84.4\;m/s$$

It is acknowledged that the Tsiolkovsky rocket equation assumes an ideal vacuum with no gravitational or aerodynamic losses, and therefore represents a theoretical upper bound on achievable velocity increment. The simplified physics-based model presented in Section 9.5, which incorporates thrust, gravity, and a constant drag coefficient, provides a more realistic first-order estimate of 221.6 m maximum altitude. The deviation between theoretical (363 m), simulated (215 m), and measured (135 m) results highlights the dominant role of aerodynamic drag, motor variability, and attitude-dependent losses in small-scale launch vehicles.

## 10. Conclusions and Future Work

### 10.1. Summary of Findings

From the perspective of sensor-system development, the principal outcome is the experimental assessment of an integrated low-cost acquisition and telemetry architecture under repeated dynamic flight conditions. The progression from 1 Hz to 100 Hz sampling demonstrated the importance of acquisition rate for resolving transient acceleration and velocity events, while the loss of the Wi-Fi link confirmed the necessity of independent onboard storage. The experiments also exposed limitations associated with one-time sensor calibration, sequential software polling, the absence of hardware-level synchronization, and unsuccessful GPS acquisition. These findings define the principal requirements for the next system iteration: pre-flight calibration, hardware timestamping, improved inter-sensor synchronization, long-range telemetry, and more reliable satellite positioning. This work demonstrated the feasibility of developing a low-cost, 3D-printed model rocket with integrated onboard telemetry and reusable flight capability. The Hermes rocket successfully collected high-frequency flight data, reaching up to 100Hz in Flight 4, enabling detailed post-flight analysis of its dynamics.

Comparison with OpenRocket simulations showed consistent deviations in performance, particularly in apogee, acceleration, and total flight duration. Across all flights, the simulated apogee was not achieved experimentally, indicating that simplified aerodynamic assumptions in OpenRocket do not fully capture real-world flight behavior under subsonic conditions.

For the final Flight 4 configuration, the measured peak velocity was approximately 14.0% below the OpenRocket prediction, whereas the apogee and time-to-apogee deviations were approximately 37.2% and 11.0%, respectively (Table 8). These deviations are attributed to a combination of motor performance variability, aerodynamic losses, and structural inefficiencies. Similar trends have been reported in prior studies using Klima D9-5 engines, reinforcing the presence of inherent variability in small solid rocket systems.

The study further validated the use of analytical modelling approaches by comparing experimental results with theoretical predictions derived from the Tsiolkovsky rocket equation and energy conservation methods. The flight computer software and data analysis pipeline were developed with the assistance of AI tools. All design files, scripts, telemetry data, and analysis resources are publicly available in the project repository at https://github.com/Filpass/Hermes-Rocket-Project (release v1.0, commit 75987c8, accessed on 20 July 2026). The repository includes raw sensor logs for each flight, the Python scripts used for telemetry processing and figure generation (Section 4.5), the OpenRocket simulation files, and CAD files for the structural components. Instructions for reproducing the plots and tables in this manuscript are provided in the repository README.

### 10.2. Limitations and Challenges Faced

The primary limitation of the project was the restricted internal volume of the rocket, driven by cost constraints and the use of clustered low-thrust motors. This limited available space for avionics integration, camera mounting, wiring management, and thermal protection against ejection charge exposure.

The current design does not explicitly account for an engine-out scenario, in which asymmetric ignition or premature burnout of one motor in the three-motor cluster would produce an off-axis thrust component, inducing an unmodeled rolling or pitching moment. This failure mode was not analyzed in the present stability assessment and is identified as a limitation of the current design.

Additional challenges included limited prior experience with embedded software development and several hardware failures during early testing phases, including Raspberry Pi failure in Flight 1 and structural separation of the nose cone in Flight 2. These failures directly informed subsequent design iterations and system improvements.

### 10.3. Recommendations for Future Work

Future developments should focus on improving avionics reliability and flight performance prediction accuracy. Key recommendations include:

- Integration of a dedicated GPS antenna system to improve satellite acquisition reliability.
- Implementation of a long-range telemetry system independent of Wi-Fi connectivity constraints.
- Structural enhancements such as deployable landing legs to reduce impact loads during recovery.
- Investigation of active or passive control surfaces on fins to improve aerodynamic stability.
- Refinement of simulation fidelity through experimentally derived drag coefficients and incorporation of higher-fidelity aerodynamic modelling techniques such as Computational Fluid Dynamics (CFD), particularly for turbulent wake prediction and low-speed flow regimes.
- Analysis of engine-out failure scenarios in clustered motor configurations, including worst-case moment estimation and possible mitigation strategies.

### 10.4. Potential Applications of the Hermes Rocket

The Hermes rocket platform demonstrates strong potential as a modular, low-cost testbed for aerospace education and experimental avionics development. Its open-source architecture enables extensions in areas such as trajectory optimization and autonomous recovery mechanisms. The system is particularly suitable for iterative prototyping and verification of flight software under real atmospheric conditions.

## Figures and Tables

**Table 1:** Comparison of the Hermes platform with representative low-cost model-rocketry flight computers and telemetry systems.

Platform	Sensors	Sampling Rate	Telemetry	Storage	Open-Source
Rohini et al. [2]	None (thrust-stand motor test only)	—	No	No	No
Brown et al. [3]	TeleMega (IMU, GPS, baro.); custom Arduino shield	—	Yes	Yes	No
Telaak & De Backer [4]	MPU-6050 IMU, MPL3115 baro., L76-M33 GPS	—	Yes (LoRa)	Yes	Yes
Binz et al. (CATS Vega) [5]	LSM6DSO32 IMU, MS5607 baro., GNSS	100 Hz	Yes (>10 km)	Yes	Yes
Moschidis & Bithas [6]	BMP180 baro., MPU6050 IMU	—	No	Yes (SD)	No
Hermes (this work)	ICM-20948 (9-axis IMU), DPS310 baro., GPS, camera	1–100 Hz	Yes (Wi-Fi)	Yes (microSD)	Yes

**Table 2:** Comparison of candidate 3D-printing materials.

Material	Density (g/cm^3^)	$T_{g}$ (°C)	Tensile Strength (MPa)	Impact Resistance	Limitations
PLA	1.24	55–60	50–60	Low, brittle failure	Low thermal resistance and brittle behavior
PETG	1.27	80–85	45–53	Moderate–high	Slightly higher mass and print complexity compared with PLA
ABS	1.05	∼105	40–50	Good	Warping tendency and hazardous printing emissions
Carbon Fiber Nylon	1.1–1.3	50–90 (matrix-dependent)	60–90+	High	High cost and increased manufacturing complexity

**Table 3:** Key simulation assumptions in OpenRocket and associated sources of uncertainty.

Category	Assumption	Effect on Accuracy
Geometry	Rigid, axisymmetric body model	Neglects deformation and assembly imperfections
Aerodynamics	Empirical drag decomposition	Limited fidelity in turbulent or transitional flow regimes
Propulsion	Idealized thrust curves	Ignores temperature-dependent and manufacturing variations
Atmosphere	Standard atmospheric model	Excludes wind shear and local environmental effects

**Table 4:** System requirements and verification methodology.

ID	Requirement	Verification Method
R-1	Minimum sensor sampling rate ≥ 1 Hz (initial requirement), increased to ≥100 Hz in final configuration	Data acquisition benchmark test
R-2/R-3	Continuous data and video storage over flight duration	Logging verification test (from sensor sampling to onboard storage confirmation)
R-4	Successful parachute deployment under simulated ejection conditions	Ejection system functional test
R-5	Autonomous system operation ≥ 15 min	Power endurance test
R-6	Static stability margin ≥ 1.5 Cal	Analytical calculation (OpenRocket) and swing test verification

**Table 5:** Flight Conditions and Environmental Parameters.

Parameter	Flight 1	Flight 2	Flight 3	Flight 4
Launch Date	5 April 2025	18 April 2025	4 May 2025	11 May 2025
Launch Site Elevation	35 m	2 m	175 m	19 m
Temperature	19 °C	17 °C	22 °C	15 °C
Atmospheric Pressure	994.47 hPa	1012.55 hPa	991.93 hPa	1015.64 hPa
Wind Speed	15 km/h	7.2 km/h	11 km/h	7 km/h

**Table 6:** Faults identified, corrective modifications implemented, and verified outcomes across the four flight tests.

Flight	Apogee	Fault Observed	Modification Implemented	Verified Outcome
Flight 1	132 m	Avionics-bay temperature rose 7.5 °C during ejection	Added thermal barrier in lower avionics compartment	Flight 2 temperature rise reduced to an increase of 0.15 °C
Flight 2	126 m	(i) Power switch caused logging termination (ii) nose cone separated near apogee	(i) Removed power switch (ii) redesigned shock cord attachment	Flight 3 logging continued for full flight (∼42 s)
Flight 3	143 m	Transient ∼20° pitch deviation near apogee	Improved fin alignment and sampling increased to 100 Hz	Flight 4 showed no comparable pitch excursion
Flight 4	135 m	Apogee remained below simulated prediction (215 m)	Not addressed in this campaign, motor limitations	Identified as future work (Section 10.3)

**Table 7:** Comparison of Flight 4 predictions from the simplified Python model and OpenRocket simulation against measured performance across all four flights.

Metric	Python Model (Fl. 4)	Open Rocket Sim. (Fl. 4)	Flight 1 Meas.	Flight 2 Meas.	Flight 3 Meas.	Flight 4 Meas.
Maximum altitude	221.6 m	215 m	132 m	126 m	143 m	135 m
Maximum velocity	56.44 m/s	58.16 m/s	42 m/s	60 m/s	68 m/s	50 m/s
Time to apogee	7.85 s	7.38 s	6.1 s	6.26 s	4.34 s	6.57 s

**Table 8:** Quantitative comparison between OpenRocket simulation and measured Flight 4 performance.

Metric	OpenRocket	Measured Flight 4	Absolute Error	Relative Error
Apogee	215 m	135 m	80 m	−37.2%
Peak velocity	58.16 m/s	50 m/s	8.16 m/s	−14.0%
Time to apogee	7.38 s	6.57 s	0.81 s	−11.0%

## Data Availability

The data presented in this study, including raw sensor logs, telemetry-processing scripts, OpenRocket simulation files, and CAD files, are openly available in the project repository at https://github.com/Filpass/Hermes-Rocket-Project (release v1.0, commit 75987c8, accessed on 20 July 2026).

## References

[1] Stine G.H., Stine B. (2004). Handbook of Model Rocketry.

[2] Rohini D., Sasikumar C., Samiyappan P., Dakshinamurthy B., Koppula N. (2022). Design & analysis of solid rocket using open rocket software. Mater. Today Proc..

[3] Brown W., Wiesneth M., Faust T., Huynh N., Montalvo C., Lino K., Tindell A. (2018). Measured and simulated analysis of a model rocket. Proc. Inst. Mech. Eng. Part G.

[4] Telaak J.T., De Backer W. (2023). Designing an Arduino-based Rocket Flight Computer for Embedded Systems Education. Proceedings of the AIAA Regional Student Conferences.

[5] Binz J., Stojoski N., Jost L. (2025). CATS: Empowering the Next Generation of Rocket Scientists through Educational Flight Computers. arXiv.

[6] Moschidis P., Bithas P.S. Arduino Rocket Flight Computer. Proceedings of the 2022 Panhellenic Conference on Electronics & Telecommunications (PACET).

[7] Sutton G.P., Biblarz O. (2017). Rocket Propulsion Elements.

[8] Halliday D., Resnick R., Walker J. (2013). Fundamentals of Physics.

[9] National Aeronautics and Space Administration Four Forces on a Model Rocket. https://www.grc.nasa.gov/www/k-12/VirtualAero/BottleRocket/airplane/rktfor.html.

[10] Wawryniuk Z., Brancewicz-Steinmetz E., Sawicki J. (2024). Revolutionizing transportation: An overview of 3D printing in aviation, automotive, and space industries. Int. J. Adv. Manuf. Technol..

[11] MarkForged (2024). PLA vs. ABS vs. Nylon. https://markforged.com/resources/blog/pla-abs-nylon.

[12] Slump G. (2021). PLA vs. ABS vs. PETG: The Differences. https://all3dp.com/2/pla-vs-abs-vs-petg-differences-compared/.

[13] Adafruit Adafruit Industries, Unique & Fun DIY Electronics and Kits. https://www.adafruit.com.

[14] ThrustCurve.org Klima D9 Motor Data. https://www.thrustcurve.org/motors/Klima/D9/.

[15] Niskanen S. (2009). Development of an Open Source Model Rocket Simulation Software. https://openrocket.sourceforge.net/thesis.pdf.

